# Data-Driven Calibration and Reference-Free Drift Detection for Multi-Modal Sensor Systems

**DOI:** 10.3390/s26217016

**Published:** 2026-11-06

**Authors:** Kishor Arvind Mohandas Sivakumar, Laurent Segers, Bruno da Silva, Argha Sen, Sujay Narayana, R. R. Venkatesha Prasad

**Affiliations:** 1 Electrical Engineering, Mathematics and Computer Science Department, TU Delft, 2600AA Delft, The Netherlands; k.a.mohandassivakumar@student.tudelft.nl; 2 Department of Electronics and Informatics, Vrije Universiteit Brussel, 1050 Brussels, Belgiumbruno.da.silva@vub.be (B.d.S.); 3 Department of Computer Science and Engineering, Indian Institute of Technology Kharagpur, Kharagpur 721302, India; arghasen10@gmail.com; 4 Skynetics Pvt. Ltd., Bangalore 560003, India

**Keywords:** calibration, calibration transfer, domain adaptation, drift detection, low-cost sensors, multi-modal sensing, environmental monitoring

## Abstract

Low-cost multi-modal sensors make high-resolution exposomic and urban-health monitoring affordable, but their data are only as useful as they are trustworthy. Measurement bias, cross-sensitivity, temporal drift and domain shift between devices and sites erode reliability, and reference instruments are rarely available for every sensor. We present a two-part reliability workflow for this realistic setting, in which reference data exist for only a subset of sensors, and evaluate it on five custom multi-modal devices deployed at two Brussels sites (9 to 68 days of data per device). For calibration, we benchmark Multiple Linear Regression, Elastic Net, Random Forest, Gradient Boosting and a Domain Adversarial Neural Network (DANN) for O_3_, NO_2_, PM_2.5_ and PM_10_ on the source device and on other devices at the same site and at the other site using MAE, RMSE, $R^{2}$, bias, device-balanced aggregation and block-bootstrap intervals. Tree ensembles achieve the lowest source-device errors for most pollutants, yet for PM_10_, they lose their advantage over the uncalibrated sensor when transferred, a caution for any deployment that calibrates on one unit. DANN attains the highest mean Calibration Transfer Ratio in the transfer settings, although with the available device pairs, the differences are not statistically significant. For modalities without a reference, a cross-sensor residual monitor combined with statistical detectors reliably flags injected gradual drift in sensors that can be reconstructed from the others (UV bands, PM_1_, temperature, humidity and CO), while Lux, pressure and VOC remain harder. The monitor detects cross-sensor inconsistency rather than isolating the faulty sensor, and the results reflect an injected fault model, not real sensor ageing. Together, these results give a transparent, reproducible baseline and clear practical guidance for building trustworthy low-cost sensor networks.

## 1. Introduction

Non-communicable diseases (NCDs) such as cardiovascular, respiratory and metabolic disorders are among the leading causes of morbidity and hospitalisation worldwide. Increasing evidence suggests that these conditions are not solely driven by genetic predisposition or lifestyle factors but are also significantly influenced by environmental exposures. The concept of the exposome captures the totality of these exposures over an individual’s lifetime. These range from air pollution and chemical agents to light, noise and climatic conditions. Accurately quantifying and modelling these environmental influences is therefore critical for improving risk prediction and enabling preventive healthcare strategies [1,2].

The ENACT project aims to develop a predictive exposomic risk score by integrating large-scale retrospective health data with environmental exposure information across multiple European populations. A key component of this effort is the deployment of multi-sensor environmental monitoring devices in pilot cities, which collect real-time data on air quality, atmospheric conditions and other environmental stressors [3]. These data streams enable the transition from population-level risk models to more personalised and context-aware assessments that reflect the actual exposures individuals experience in their daily environments.

However, the reliability of such sensor-driven approaches depends heavily on the accuracy and stability of the measurements. Low-cost environmental sensors, while scalable and suitable for large deployments, are prone to issues such as measurement bias, cross-sensitivity and temporal degradation due to ageing or changing operating conditions [4]. In addition to gradual changes over time, sensors may also exhibit abrupt faults due to hardware malfunctions, communication errors or environmental interference [5].

Multi-modal sensing systems introduce additional challenges beyond those encountered in single-sensor calibration tasks. Different sensing principles result in different measurement biases, cross-sensitivities and ageing behaviour. Electrochemical gas sensors may drift due to chemical degradation [6], and optical sensors are highly sensitive to illumination conditions [7], while particulate sensors are affected by humidity and particle composition [8]. Consequently, reliability management strategies that perform effectively for one modality may not generalise across others. This motivates the need for unified frameworks that can handle diverse sensing behaviours on a single deployment platform.

This paper addresses these challenges through a unified framework specifically designed for multi-modal sensing systems used in the ENACT project. Conventional studies often rely on isolated sensing modalities or assume continuous access to reference instrumentation. In contrast, this work investigates calibration and drift detection across a diverse array of sensors operating simultaneously under real-world deployment conditions.

A central research challenge in such systems is that reference ground truth is only partially available. While certain pollutants such as NO_2_, O_3_, and PM can be calibrated against nearby regulatory monitoring stations, many auxiliary modalities—including optical, VOC, CO_2_, and environmental sensors—lack equivalent reference infrastructure. This creates a partially supervised sensing problem where calibration, cross-modal consistency, and drift monitoring must be jointly considered rather than treated independently. This paper investigates the following research questions:

**RQ1:** Which calibration models provide the best performance for multi-modal sensing systems?
**RQ2:** Can cross-sensor dependencies be leveraged to detect sensor drift in modalities lacking external reference measurements?
**RQ3:** How do different sensing modalities respond to device and inter-location domain shifts and temporal drift?  

RQ1 and RQ3 are answered for the four reference-covered pollutants through the calibration experiments (methods in Section 4.1, results in Section 5.1): RQ1 by the source-device evaluation and RQ3 by the intra-location (device-to-device) and inter-location (Halle ↔ Ukkel) transfer evaluations. RQ2 and the temporal-drift part of RQ3 are answered for the sensors without references through the reference-free drift-detection experiments (methods in Section 4.2, results in Section 5.2). Because the reference-free modalities cannot be evaluated for geographical transfer, RQ3 is addressed for them only through cross-sensor predictability and controlled synthetic drift. All answers are limited to the five devices, two locations and one injected-fault model studied here (Section 7).

The individual techniques used here (tree-based calibration, domain-adversarial training, residual modelling, CUSUM and EWMA charts) are established. The contribution of this paper is therefore not a new algorithm but a systematic side-by-side empirical evaluation of these techniques on one heterogeneous multi-modal platform, with source-device, device-to-device and location-to-location behaviour reported separately, and with an explicit statement of what a reference-free residual monitor can and cannot conclude.

### 1.1. Sensing Platform and Deployment

**Figure 1:**
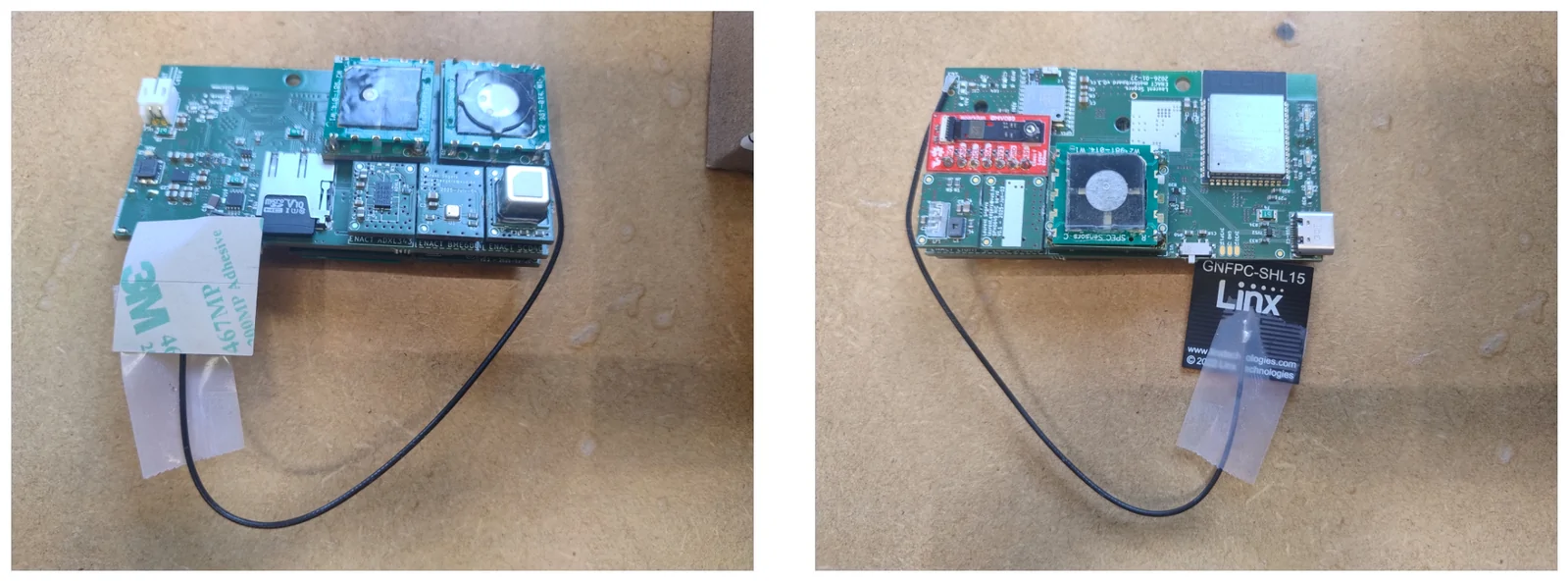
The ENACT custom-designed multi-sensor environmental monitoring device used in this study.

The custom monitoring device (Figure 1) integrates a suite of sensors to capture a wide array of environmental stressors. The device includes the following sensors:

**VEML7700:** ambient light (lux);
**AS7331:** spectral light sensing (UVA,UVB,UVC);
**BMV080:** particulate matter ($PM_{1},PM_{2.5},PM_{10}$);
**SPH0645:** acoustic measurements (microphone, not used in this study);
**BME680:** temperature, humidity, pressure and volatile organic compounds (VOC);
**SCD40:** $CO_{2}$ concentration, temperature and humidity;
**Other gas sensors:** O3 (110-407), NO2 (110-508) and CO (110-109).

Sensory data collection is managed by an ESP32-S3 microcontroller programmed via the Espressif extension for Visual Studio Code (version 1.108). The device wakes up every 5 min to take several consecutive samples before returning to deep sleep. Utilising deep sleep conserves battery power and minimises thermal heating of the platform, which could otherwise unnecessarily distort sensor readings. The configuration of the selected sensors enables simultaneous measurement of optical, particulate, gaseous and environmental parameters relevant to exposomic analysis.

**Figure 2:**
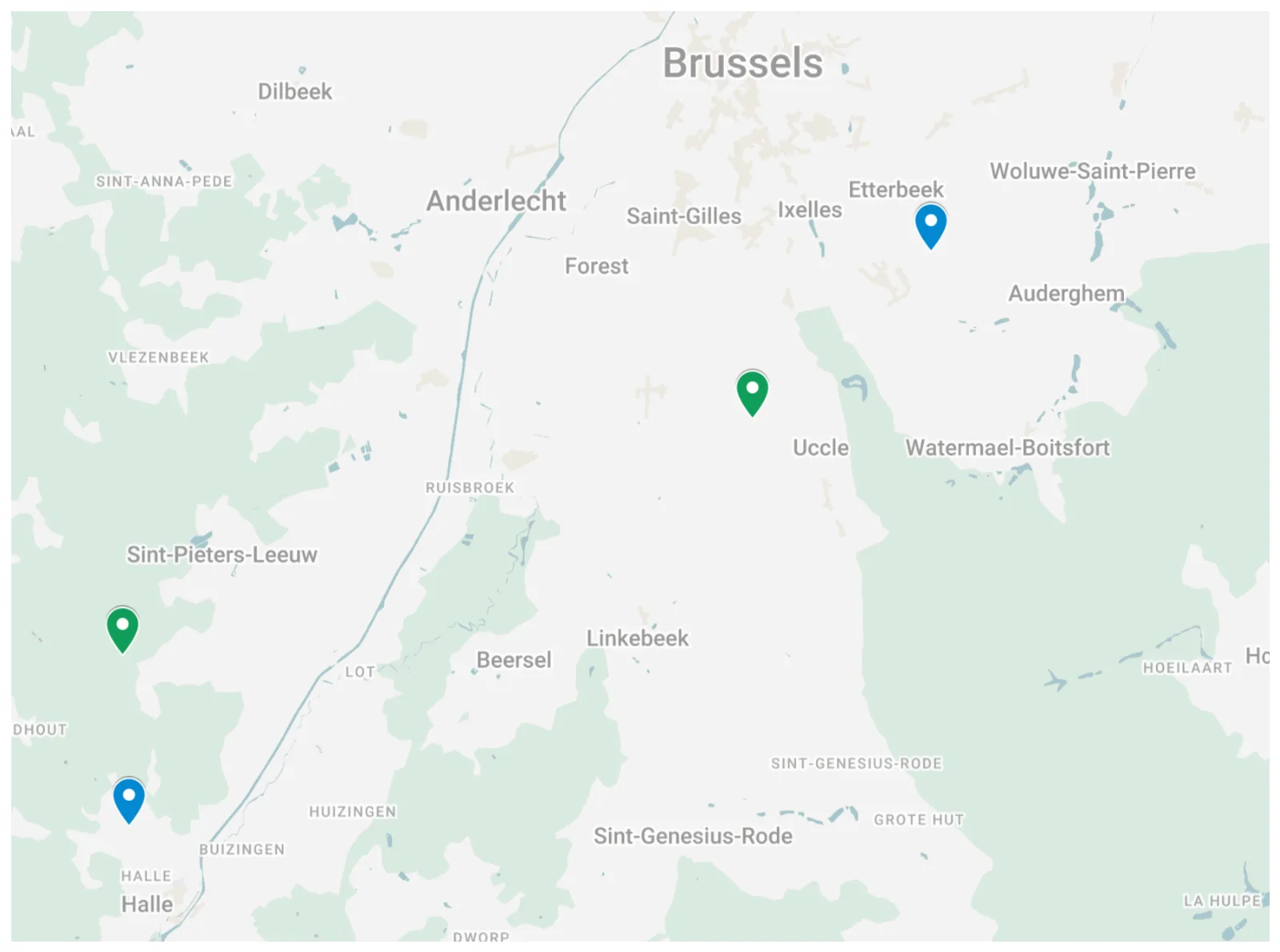
Geographical deployment of sensing nodes (blue) and corresponding reference stations (green) in Brussels.

Five of these devices were deployed in a pilot study across Brussels, Belgium. To test how the models behave under domain shift, the deployment was distributed across two distinct locations: two devices were positioned in Halle, and another three devices were positioned in Ukkel. Reference measurements were sourced from the nearest regulatory monitoring stations operated by Irceline, the Belgian Interregional Environment Agency. Figure 2 illustrates the spatial distribution of the deployed sensor nodes (represented by blue pins) relative to their nearest high-precision Irceline monitoring stations (represented by green pins).

These reference stations provide continuous ground-truth data for the primary target pollutants: O_3_, NO_2_, PM_10_ and PM_2.5_. Using these reference measurements, supervised calibration models were trained to map the relationship between raw, uncalibrated sensor signals and the true environmental concentrations.

For sensors without direct reference data (Lux, UV, CO_2_, CO, PM_1_, VOC, temperature, humidity and pressure), this study leverages cross-sensor relationships to train the models. Moreover, redundancy is built into the device for certain key variables, such as temperature and humidity, which are measured by both the BME680 and SCD40 sensors. These redundant measurements were also leveraged for model training. Residuals are generated as the difference between the raw sensor value and the model predictions. These residuals are monitored over time to detect sensor drift.

This experimental setup enables a rigorous evaluation of model performance under varying environmental conditions and across different devices and locations. By design, the framework allows us to first train and validate our calibration and drift detection methodologies on a single, isolated device. We then systematically evaluate the model’s cross-device transfer and cross-location transfer performance. All data were time-synchronised and preprocessed to handle missing values, outliers, and sensor anomalies prior to model training; the preprocessing steps are described in Section 3.2.

### 1.2. Objectives and Contributions

The primary objective of this work is to develop a scalable reliability framework for multi-modal sensing systems operating under partially supervised conditions, where reference measurements are available for only a subset of sensing modalities. The specific contributions of this paper are as follows:

**Multi-Modal Sensor Calibration:** We compare five calibration models for NO_2_, O_3_, PM_2.5_ and PM_10_ using MAE, RMSE, $R^{2}$ and bias, with device-balanced aggregation and block-bootstrap confidence intervals.
**Domain Shift Analysis:** We investigate calibration transfer to other devices at the same location and to the other location, and report absolute target-domain error alongside the Calibration Transfer Ratio.
**Multi-Modal Drift Detection:** For auxiliary sensors lacking external ground truth, we evaluate a reference-free monitor that detects gradual cross-sensor inconsistency through residual analysis and a suite of statistical tests, using injected synthetic drift.
**Curated Multi-Modal Field Dataset and Open Code:** We describe a curated multi-modal field dataset of gaseous, particulate, optical and environmental observations from five devices, and we release the preprocessing, calibration and drift-detection code. The dataset itself is not openly archived; its availability is stated in the Data Availability Statement.  

The remainder of this paper is organised as follows: Section 2 reviews related work; Section 3 describes the system and the data preprocessing pipeline; Section 4 describes the calibration and drift-detection methods; Section 5 presents the results (RQ1 and RQ3 in Section 5.1, RQ2 and the drift part of RQ3 in Section 5.2); Section 6 discusses the answers to RQ1 to RQ3 and their implications for the ENACT project; Section 7 states the limitations; and Section 8 concludes with future work.

## 2. Related Works

Research on low-cost environmental sensing relevant to this work can be divided into three closely related areas: reference-based sensor calibration, transfer of calibration models between sensing domains, and fault or drift detection when continuous reference measurements are unavailable. Surveys of environmental sensor networks distinguish between reference-based, partially blind and blind calibration, and emphasise that calibration performance depends not only on the regression method but also on sensor technology, environmental conditions and the availability of reference measurements [4,9].

Table 1 summarises representative studies related to the calibration, calibration-transfer and fault-detection problems considered in this work.

### 2.1. Reference-Based and Multi-Sensor Calibration

Reference-based calibration typically learns a mapping from low-cost sensor measurements to a higher-quality reference while incorporating environmental variables that influence the sensor response. Early work, therefore, relied heavily on linear regression and environmental compensation, while more recent studies increasingly compare regularised and nonlinear machine learning models. Maag et al. used multiple least squares to quantify the influence of target pollutants, interfering pollutants, temperature and humidity in cross-sensitive air-quality sensor arrays [27]. Pastorio et al. similarly evaluated linear regression, support vector regression and Random Forest for low-cost particulate matter sensors, using temperature and humidity as auxiliary variables [8]. These studies illustrate the importance of accounting for information beyond the primary sensor channel when environmental and cross-sensitivity effects are significant.

Recent sensor calibration studies, particularly for air quality sensors, use a combination of linear and nonlinear models. Taştan evaluated linear regression together with decision trees, Random Forest, KNN, AdaBoost, Gradient Boosting, SVM and SGD for a heterogeneous platform measuring PM_2.5_, CO_2_, temperature and humidity [28]. Mohanasundaram et al. used Elastic Net for feature selection and combined that with SVM, RF and GB for photovoltaic energy prediction [13]. Related environmental prediction studies have also evaluated combinations of linear models, Elastic Net, tree ensembles, SVMs and neural networks across meteorological and optical variables [12,29]. Collectively, these results motivate comparing both interpretable linear approaches and nonlinear models rather than assuming that greater model complexity will consistently provide better calibration.

The relevance of multi-modal compensation is particularly clear for sensing technologies that are affected by other environmental variables. Abdulnasser et al. modelled the influence of temperature and humidity on metal-oxide VOC sensors and used regression-based correction to compensate for these effects [6]. Nava-Pintor et al. combined low-cost light measurements with temperature and humidity and compared linear, polynomial, RF and SVR models [7]. More recently, Mahajan and Helbing proposed dynamic PM_2.5_ calibration based on a trust score derived from accuracy, stability, responsiveness and agreement with neighbouring sensors, demonstrating another way in which information from multiple sensing nodes can support calibration when reference coverage is limited [12]. These works support the use of heterogeneous sensor information as the explanatory context and show that its usefulness depends on the physical relationships among the measurements.

### 2.2. Calibration Transfer and Domain Shift

High calibration accuracy on the device or location used for training does not necessarily imply that the model will remain accurate after deployment elsewhere. Transfer introduces both unit-to-unit differences between nominally identical sensors and changes in the distributions of pollutants and environmental variables. Nowack et al. [17] examined this problem for NO_2_ and PM_10_ sensors and found that nonlinear models could perform strongly at the calibration site but lose their advantage after relocation; in some transfer conditions, regularised linear models were more robust because nonlinear models were required to extrapolate beyond their training distribution. This distinction between source accuracy and transferability is central when a calibration model is intended for deployment on other sensor nodes.

Recent large-scale studies reinforce the importance of the deployment domain. Hayward et al. [10] evaluated more than 470,000 correction configurations for NO_2_, O_3_, PM_2.5_ and PM_10_ and found that device type and deployment environment could influence performance more strongly than the choice of regression algorithm itself. In [18], the authors evaluated PM_2.5_ calibration transfer across 22 monitoring sites and showed that successful transfer was related more closely to similarities in aerosol characteristics and source composition than to geographical distance alone. Abu-Hani et al. likewise observed stronger transfer between sensor units operating at the same site than between geographically different sites for electrochemical NO and NO_2_ sensors [15]. Together, these findings motivate treating transfer between devices in the same environment separately from transfer between different geographical environments.

Consequently, reducing the amount of target-domain reference data required for transfer has received considerable attention. Cheng et al. considered a deployment containing approximately 1000 low-cost PM_2.5_ sensors but reference information only from a small subset of nodes. Their in-field calibration-transfer method exploits similarity between calibrated and uncalibrated sensor distributions to propagate calibration through the network [16]. This approach is attractive for large deployments, although it primarily addresses a homogeneous PM-sensor network.

Domain adaptation provides an alternative by explicitly learning representations that are less dependent on the source domain. Domain Adversarial Neural Networks (DANNs) achieve this by training a label predictor together with a domain classifier connected through a gradient-reversal layer, encouraging the learned representation to remain predictive of the target quantity while suppressing domain-specific information [30]. Dey et al. [14] applied a related semi-supervised domain-adaptation approach to low-cost sensor regression using labelled source-domain measurements and unlabelled target-domain data, with additional weighting to account for differences between the source and target distributions. These approaches are particularly relevant where a target device can provide raw measurements but cannot be continuously co-located with a reference instrument.

### 2.3. Temporal Drift, Fault Detection and Reference Scarcity

Calibration transfer addresses differences between deployment domains, whereas temporal drift concerns changes in sensor behaviour during continued operation. Long-term evaluations demonstrate that these changes can be significant. The QUANT study evaluated 43 commercial air-quality devices, comprising 119 gas sensors and 118 PM sensors, over three years and reported substantial differences in performance and stability across technologies and deployment environments [31]. Gäbel and Hertig further investigated recalibration frequency for low-cost O_3_ and PM_2.5_ sensors over a one-year deployment and showed that periodic recalibration was necessary to maintain quantitative performance [11]. Continuous reference co-location, however, is generally impractical for large distributed deployments, motivating methods that infer sensor health from the behaviour of the sensing system itself.

Residual-based fault detection is particularly relevant in this setting. The general principle is to learn the expected behaviour of a measurement during healthy operation and monitor the difference between the measured and predicted values. Xu et al. [20] combined Random Forest normal-behaviour modelling with CUSUM monitoring of the resulting residuals for wind-turbine sensor fault detection. CUSUM accumulates persistent deviations and is therefore well suited to changes that may be difficult to identify from individual residual samples. Parzinger et al. [23] adopted a broader statistical approach, generating residuals from predictive models and evaluating them with multiple sliding-window statistical tests. The outputs of these tests were subsequently combined using decision rules to distinguish normal and faulty operation. These studies provide direct precedent for applying statistical change-detection methods to prediction residuals rather than to the raw measurement itself.

Several studies also exploit redundancy among sensors to reconstruct the expected signal. Al-Zuriqat et al. used multiple co-located temperature sensors and trained an ANN to predict individual sensor measurements from the remaining sensors, allowing deviations between predicted and observed values to indicate faults [25]. Chahine and Noura use a closely related virtual-sensor formulation: LightGBM models estimate individual temperature sensors from correlated measurements, after which prediction residuals are standardised using statistics from healthy operation and evaluated using a threshold-based detector [24]. This is particularly similar to reference-free monitoring in a multi-sensor system, where the expected measurement is obtained from other sensing channels rather than from an external reference.

Residual-based approaches have also been explored using different predictive models and decision statistics. Hsu et al. compared residual-based fault indicators generated using autoencoder models [26], while Narzary and Veluvolu proposed an index-based residual method for detecting multiple sensor faults [32]. Specifically, in environmental monitoring, Lin et al. proposed a two-stage framework for identifying malfunctioning PM_2.5_ sensors in a large-scale air-quality network, using learned temporal behaviour followed by threshold-based detection [21]. Rollo et al. instead combined temporal, multivariate and environmental-context anomaly detectors for NO, NO_2_ and O_3_ measurements and fused their decisions through majority voting [33]. Although their detectors operate on raw observations rather than regression residuals, the work provides relevant evidence that complementary detectors can be combined to improve anomaly identification in environmental sensing.

A practical difficulty shared by these studies is the scarcity of labelled examples of genuine sensor degradation. Controlled or synthetic fault injection is therefore frequently used to evaluate whether detectors respond to known abnormal behaviour. This allows the detector response and false-alarm behaviour to be quantified even when long-term labelled drift records are unavailable. The literature consequently supports the main components of residual-based monitoring used in this work: learning healthy relationships between sensors, generating prediction residuals, applying statistical detectors to persistent residual changes and combining complementary detection mechanisms.

### 2.4. Research Gap

Existing work provides substantial evidence for each of the individual components required for reliable low-cost sensing. Supervised calibration studies show that environmental compensation and nonlinear models can substantially alter calibration performance, while transfer studies demonstrate that models successful on their source domain can degrade when applied to different devices or environments. Domain-adaptation methods reduce this dependence on labelled target data, and residual-based fault-detection studies show that sensor redundancy can be used to monitor measurements when a direct reference is unavailable.

However, these problems are usually investigated separately. Calibration and calibration-transfer studies predominantly focus on a single pollutant family or on networks of similar sensors, whereas the closest residual-based fault-detection approaches are generally evaluated on homogeneous sensor groups or on industrial and building-monitoring systems. Environmental anomaly-detection studies, meanwhile, often identify abnormal raw observations rather than jointly modelling the health of heterogeneous sensing modalities.

The present work addresses these three problems within a single multi-modal platform. For pollutants with regulatory ground truth, multiple linear and nonlinear models are compared for calibration. Their performance is evaluated in the source domain, as well as how well they transfer between devices and between locations. For sensing modalities without continuous reference measurements, each sensor is predicted from the remaining measurements using the same models, and the resulting residual is monitored with complementary detectors. Their outputs are combined through a sensor-specific weighted ensemble and persistence rule to produce the final detector. This provides a common framework for examining how sensing modality, cross-sensor redundancy and deployment-domain changes influence both calibration transferability and reference-free drift detection.

#### 2.4.1. Closest Prior Work

The studies in Table 1 differ in sensor type, ground truth, task and fault model, so the table supports a qualitative statement of the gap only. The closest studies for calibration and transfer are Hayward et al. [10], Abu-Hani et al. [15] and Malyan et al. [18]; the closest redundancy-based residual monitors are Al-Zuriqat et al. [25] and Kullaa [22]. To our knowledge, none of them evaluates calibration transfer and reference-free monitoring together on one heterogeneous platform. The contribution of this paper is this combined systematic evaluation, not a new calibration or drift-detection algorithm.

#### 2.4.2. Fault Isolation and Simultaneous Faults

In process monitoring, isolating the faulty sensor is a separate step from detection and is addressed with structured or directional residuals: a bank of residual generators, each insensitive to a different subset of sensors, produces an alarm pattern that identifies the faulty channel, and simultaneous faults are handled by designing the pattern table for combinations of faults [34,35]. The monitor studied here builds one residual per target from all remaining sensors, so a fault in one predictor perturbs several residuals at once. It detects inconsistency but is not designed to isolate its cause. Isolation has been demonstrated for air-quality monitoring networks with PCA-based schemes [19].

#### 2.4.3. Uncertainty in the Residual Model

Residual monitoring depends on how well the healthy residual spread is known. Quantile regression forests [36] and conformal prediction [37] give prediction intervals that vary with the operating condition and need few distributional assumptions; Allka et al. used Gaussian-process predictive intervals to flag drift in IoT air-quality sensors [38]. This work uses one global standard deviation estimated on healthy data, which assumes constant residual variance; heteroscedastic residuals are a plausible cause of the high false-positive rates for CO_2_ and VOC. Deep CORAL [39] and maximum-mean-discrepancy-based adaptation [40] are alternatives to adversarial adaptation that were not evaluated here.

## 3. System Overview and Data Processing

The integrity of the exposomic risk models relies on a robust data pipeline that transforms raw sensor outputs into calibrated, reliable environmental features. This section details the data collection, preprocessing, feature engineering, and the mathematical foundations of the models employed for both calibration and drift detection.

### 3.1. Data Collection

**Figure 3:**
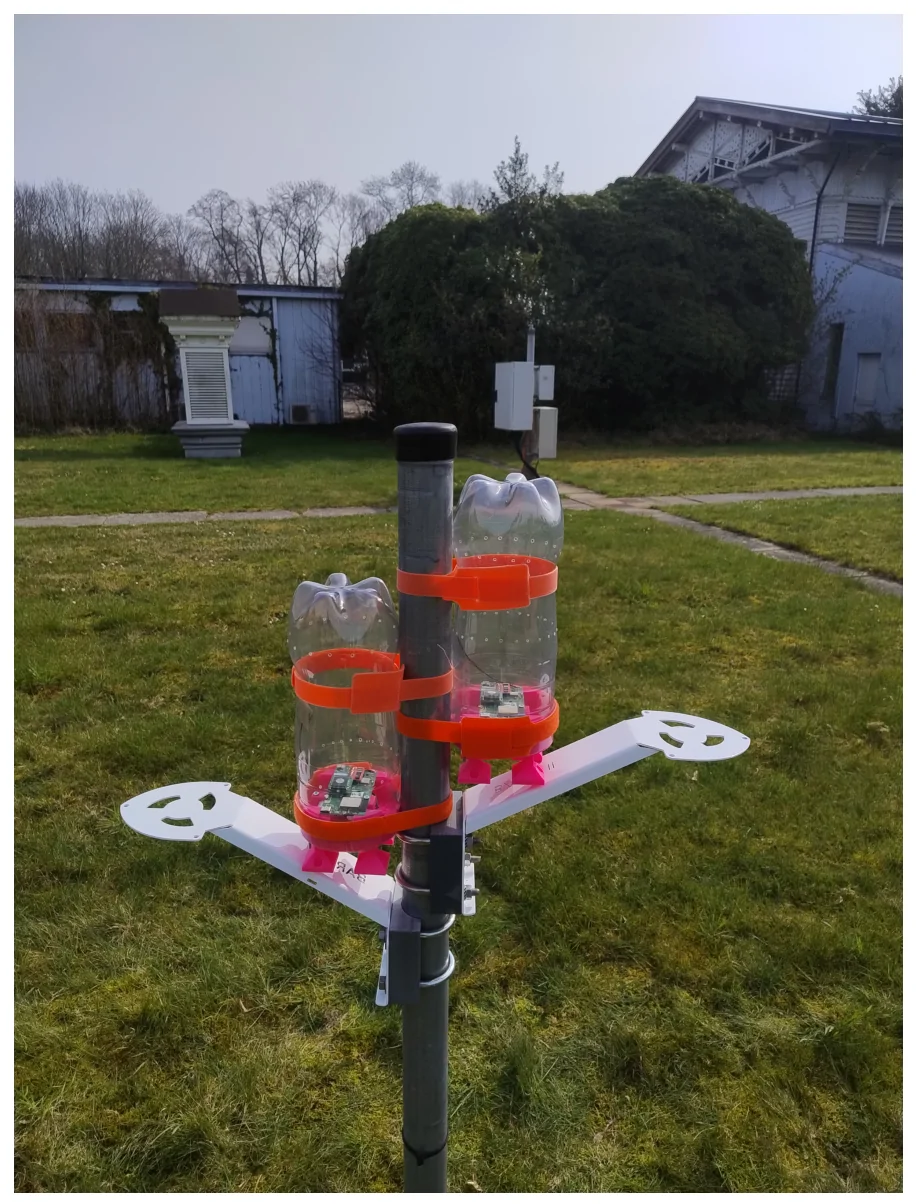
Data collection.

Five ENACT devices were deployed outdoors at two Belgian locations: two devices in Halle and three devices in Ukkel. Operating more than one device at each location creates the device combinations required for the intra-location transfer experiment, while the two locations provide the domains used in the inter-location experiment. The devices were exposed to ambient conditions in their protective enclosures, as illustrated in Figure 3.

Each continuous deployment lasted approximately two to six days, primarily because of the available battery capacity. At the end of a deployment cycle, the device was retrieved, its raw CSV files were downloaded from the SD card and the battery was charged before redeployment. The data for one device are therefore distributed across several acquisition files rather than one uninterrupted record. Concatenation had to preserve chronological order while accounting for gaps produced by retrieval, charging and redeployment.

The sensing devices normally operate using a duty-cycled acquisition scheme. Each device wakes approximately every five minutes, records two measurements separated by approximately six seconds and then returns to low-power operation. During some charging periods, measurements were recorded continuously without the five-minute wake interval. The acquisition parameters are summarised in Table 2.

The effective sampling rate is calculated from two measurements per 300 s wake cycle, corresponding to approximately 576 measurements per complete day. During charging, the wake interval was not applied and measurements could be recorded continuously at approximately one sample every six seconds, corresponding to as many as 14,400 measurements per day. Charging periods could therefore produce approximately 25 times as many observations per unit time as battery-powered operation. The time each device spent charging consequently contributes to differences in raw sample counts between devices.

The two pipelines treat this imbalance differently. In the calibration pipeline, all measurements within a clock hour are averaged into one sample, so a charging hour and a battery hour have the same weight. In the drift-detection pipeline, the native sampling rate is retained and no re-weighting was applied, so charging periods contribute more (and more strongly autocorrelated) samples to model fitting, detector tuning and alarm counting. The 15-sample persistence rule spans about 90 s of charging data and about 37 min of battery data, and the detection rates reported below are rates over samples, not over time.

The calibration and drift-detection pipelines use the same underlying measurements at different temporal resolutions. For calibration, measurements within the same clock hour are averaged to obtain a single sample per hour, matching the regulatory reference data. Drift detection instead retains the measurements at their native acquisition resolution, preserving short-term changes in sensor behaviour. The drift dataset, therefore, contains both duty-cycled measurement bursts and, where present, more densely sampled charging periods.

The resulting dataset is unbalanced across devices. The Halle devices were operated for substantially longer than the Ukkel devices and span a wider range of seasonal conditions. The five devices also do not contain an identical set of auxiliary channels. Most notably, UV-A, UV-B and UV-C measurements are missing on two out of the three Ukkel devices. CO measurements are also only available for Halle devices. Missing channels are not synthetically reconstructed before the experiments; the models use the measurements that are available on the relevant device. Consequently, some target-sensor prediction tasks differ in the amount of supporting information available. This variation is treated as a property of the deployed system and is analysed explicitly in the Lux drift-detection results.

### 3.2. Data Preprocessing

**Figure 4:**
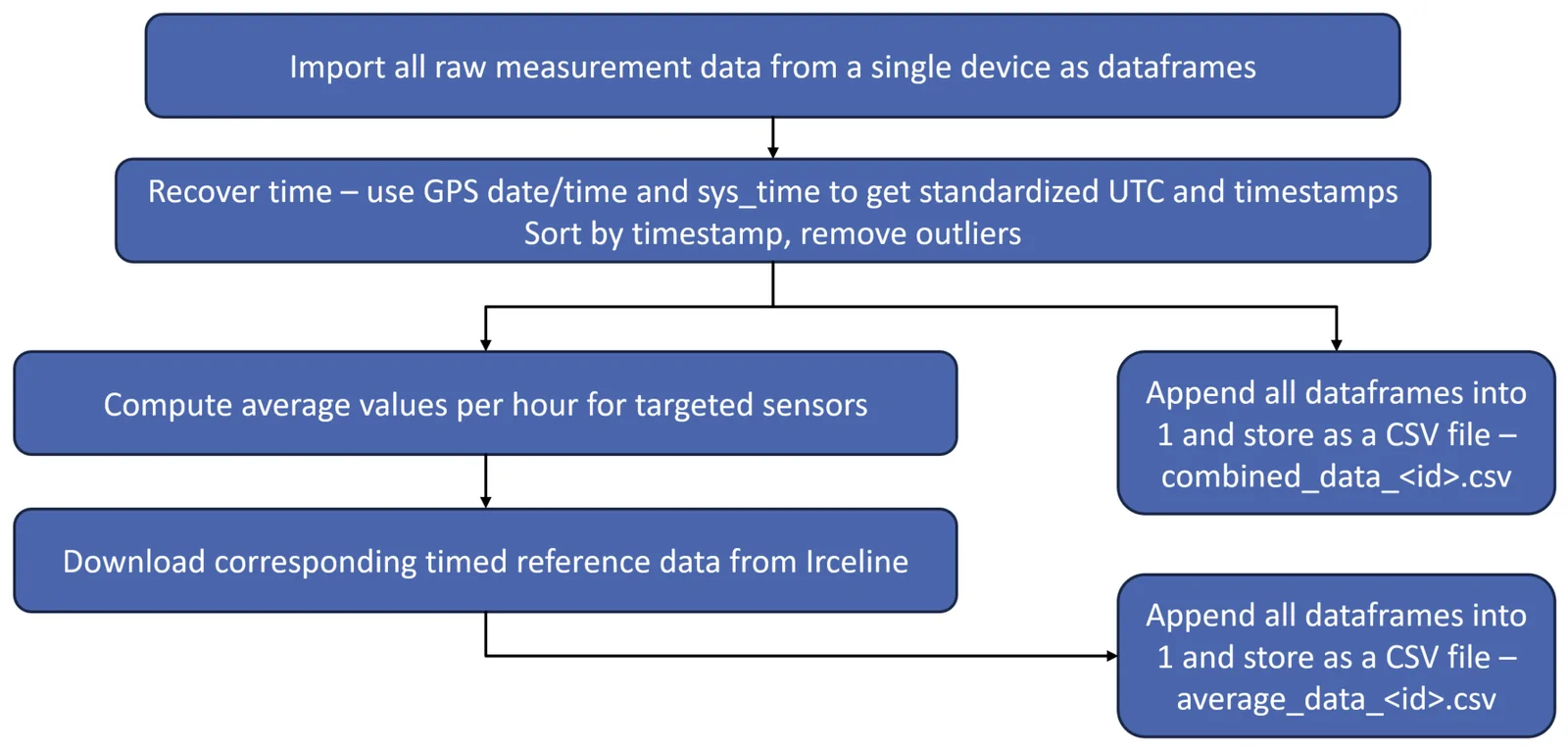
The data preprocessing pipeline, illustrating the bifurcation into high-resolution data for drift detection and hourly averaged data for reference calibration.

Raw measurements were recorded in separate CSV files for each deployment cycle. A common preprocessing pipeline was therefore applied to every device before either calibration or drift analysis. Figure 4 shows how the common sequence is divided into a reference-aligned calibration branch and a higher-resolution drift-detection branch.

The preprocessing consists of the following stages:

1. **Timestamp reconstruction and ordering:** UTC timestamps are reconstructed using the recorded GPS date and time together with the system clock. Records are placed in chronological order so that observations from successive deployment files form one device-level time series.
2. **Cleaning and consistency checks:** Invalid timestamps, duplicate records and measurements identified as physically implausible by the preprocessing rules are removed. Specifically, raw O_3_ and NO_2_ electrochemical readings that are negative or exceed 100 mV are treated as invalid and set to zero before hourly averaging; no other channel is clipped. Rows with a missing value in any model input or in the reference variable are dropped for the calibration experiments (complete-case analysis); in the calibration branch, missing values are not imputed. In the drift-detection branch, the rows with a missing target value are removed and any remaining gaps in the predictor channels are filled by forward fill followed by backward fill before the split is made. Records before 1 December 2025 are discarded.
3. **High-resolution drift detection branch:** Cleaned files from repeated deployments are concatenated for each device. This is stored as combined_data_<id>.csv and is used as the dataset for drift detection experiments.
4. **Reference-aligned calibration branch:** Device measurements are aggregated into hourly intervals (arithmetic mean of all samples in the clock hour, timestamped at the start of the hour in UTC) and aligned by timestamp with the available IRCELINE regulatory measurements. This is stored as average_data_<id>.csv and is used as the dataset for supervised calibration experiments.

**Reference data and spatial mismatch.** The reference values are hourly means from the IRCELINE open SOS service in μg/m^3^. For the Halle devices, O_3_ and NO_2_ come from the Sint–Pieters–Leeuw station and PM_10_ and PM_2.5_ from the Uccle station; for the Ukkel devices, all four pollutants come from the Uccle station. Based on approximate municipality-centre coordinates (exact device coordinates were not recorded), the Halle devices are about 4 km from the Sint–Pieters–Leeuw station and about 11 km from the Uccle station, whereas the Ukkel devices are within a few kilometres of Uccle. Part of the error reported for Halle PM therefore reflects spatial representativeness rather than sensor error, and the intra- and inter-location PM results are partly confounded by the reference-station distance.

**Alignment.** For each deployment file, the IRCELINE series was requested for the span from the first to the last device hour and paired with the hourly device table in time order. Hours without a valid device or reference value are removed before modelling (complete-case analysis).

Each device therefore produces one processed calibration dataset and one processed drift-detection dataset.

### 3.3. Dataset Description

Table 3 summarises the datasets collected from the five deployed devices. The two Halle devices span approximately 68 days and provide the only sufficiently long reference-aligned records for temporal analysis. Ukkel_1 and Ukkel_2 each span approximately nine days, while Ukkel_3 spans approximately 22 days. The number of observations is not determined by deployment duration alone: it also depends on data gaps, channel availability, reference alignment, preprocessing and the amount of time for which measurements were recorded during charging. Because charging-period acquisition occurred without the five-minute wake interval, devices with more charging data can contain substantially more drift-detection samples despite having a shorter deployment span. Calibration sample counts are affected less directly because the measurements are aggregated hourly.

Calibration samples are the valid hourly device–reference pairs left after complete-case filtering. The Halle records are spread intermittently over December 2025 to July 2026 and therefore include winter, spring and early-summer conditions, whereas each Ukkel device covers a single season: Ukkel_1 and Ukkel_2 the turn of January to February 2026 and Ukkel_3 the period ending on 2 May 2026.

Environmental measurements collected close together in time share weather, emission and illumination conditions and are therefore correlated. In addition, the short Ukkel deployments cover a narrower range of conditions than the Halle records. The limited number of devices remains a separate source of uncertainty and is addressed in Section 7.

### 3.4. Feature Engineering and Normalisation

To stabilise training and improve model generalisation across different hardware nodes, several feature engineering techniques were applied:

- **Normalisation:** Inputs to MLR, Elastic Net and DANN were standardised with Z-score normalisation, and the calibration target was standardised for the classical regressors. The scaler parameters are estimated on the training partition of the source device only and are then applied unchanged to the held-out and target-device data. Tree ensembles (RF and GB) are scale-invariant and received unscaled inputs.
- **Ratios:** Two derived particulate features were used: $\log(1+{\mathrm{PM}}_{10}/{\mathrm{PM}}_{2.5})$ and $\log(1+{\mathrm{PM}}_{2.5}/{\mathrm{PM}}_{1})$.
- **Temporal Encoding:** To account for daily and seasonal cycles, hour-of-day and month-of-year values were transformed using cyclic sine and cosine functions:
  $$h_{\sin}=\sin\left(\frac{2\pi t}{T}\right),\;h_{\cos}=\cos\left(\frac{2\pi t}{T}\right)$$
  where *t* is the time index and *T* is the period (24 h for the hour encoding and 12 months for the month encoding). This provides context to the models on the cyclic nature of the measurements. Because the Ukkel deployments cover only one to three calendar months, the month encoding can act as a deployment-period identifier for those devices; its influence on the transfer results was not isolated.

### 3.5. Models and Tests Used

We compare the following combination of linear, ensemble, and adversarial models for calibration. The models are trained to predict the reference value of the sensor using the raw value, environmental parameters, and multi-modal context.

#### 3.5.1. Standard Regression Models

- **Multiple Linear Regression (MLR):** Serves as the interpretable baseline for sensor inter-relationships.
- **Elastic Net (EN):** A regularised regression that combines $L_{1}$ (Lasso) and $L_{2}$ (Ridge) penalties to handle potential multicollinearity between sensors.

#### 3.5.2. Machine Learning Models

- **Random Forest (RF) and Gradient Boosting (GB):** nonlinear ensemble methods selected for their ability to model complex nonlinear cross-sensitivities and their robustness to noise.
- **Domain Adversarial Neural Networks (DANNs):** Integrated to address geographical distribution shifts. DANNs utilise a gradient reversal layer to extract features that are discriminative for calibration but invariant to the specific device or location, facilitating better model transferability.

#### 3.5.3. Statistical Tests

The same models are used for drift detection. However, as reference data is unavailable, the models are trained to predict the raw value of the sensor using environmental parameters and multi-modal context. The residuals (predicted - actual) are monitored using the below tests to detect drift.

- **Z-score thresholding:** A flag is raised if normalised residual crosses a fixed threshold; detects large instantaneous deviations and abnormal spikes.
- **CUSUM [41]:** Accumulates deviations over time; detects persistent mean shifts caused by gradual sensor bias.
- **EWMA [42]:** Smooths residuals using exponentially weighted averaging so that persistent small deviations gradually accumulate; detects slowly varying baseline drift.
- **Rolling Mean Test:** Detects local changes in residual mean and variance caused by intermittent instability.

### 3.6. Parameter Optimisation

The optimal parameters for each model and test depend on the specific device and sensor. Hence, during training, each combination is tested using a grid search approach to find the optimal parameters. For the classical calibration models, the grid search is performed on the training partition of the source device only, with 3-fold expanding-window time-series cross-validation (scikit-learn TimeSeriesSplit) [43,44] and MAE as the criterion; the held-out source test block and the target devices play no role in this selection. For DANN, each of the four learning-rate and dropout combinations is trained once and the combination with the lowest MAE on the source training data is retained, i.e., no separate validation split is used for DANN (see Section 4.1.1). The resulting hyperparameter search grids are summarised in Table 4.

A similar approach is used to optimise the statistical tests. Here, all four tests are combined for the final output, so a weighted ensemble rule is also optimised to find the best combination. This is explained in detail in the next section. The evaluated statistical-test and ensemble configurations are summarised in Table 5.

## 4. Calibration and Drift Detection Methods

### 4.1. Supervised Calibration

For pollutants with available reference measurements (NO_2_, O_3_, PM_10_ and PM_2.5_), sensor calibration is formulated as a supervised regression problem. The objective is to learn a calibration function

$$\hat{y}=g\left(x\right),$$

where x represents a multi-modal feature vector obtained from the sensing device and $\hat{y}$ is the calibrated estimate of the reference measurement.

Unlike conventional calibration approaches that primarily rely on raw sensor measurements and environmental variables, this work additionally incorporates information from multiple sensing modalities. Each calibration model, therefore, receives a heterogeneous feature vector containing:

- **Primary measurement:** the raw output of the target sensor.
- **Environmental context:** temperature, relative humidity and atmospheric pressure, which compensate for known environmental influences on sensor response.
- **Cross-modal measurements:** observations from the remaining sensing modalities, including particulate matter, gas sensors, optical sensors and VOC measurements, allowing the model to exploit potential cross-sensitivities and inter-sensor relationships.

A separate calibration model is trained for each target pollutant, using the same input feature vector while learning the relationship between these features and the corresponding pollutant-specific reference measurement.

#### 4.1.1. Experimental Protocol

For each source device, the reference-aligned hourly samples are ordered in time and a contiguous 20% block is withheld as the source test set; the remaining 80% is used for training and model selection. For the Ukkel devices, the test block is the final 20% of the record, and for the Halle devices, it is the final 20% of each calendar month. Hyperparameters of MLR, EN, RF and GB are selected by grid search with three expanding-window time-series folds drawn only from the training part, and feature and target scaling are fitted on the training part only. No target-device data or target reference values are used for feature engineering, scaling or model selection. A trained source model is applied unchanged to the complete reference-aligned record of every other device: devices at the same location form the intra-location scenario, and devices at the other location form the inter-location scenario.

Neighbouring hours are autocorrelated, so device pairs and not individual hours are treated as the units of replication. Repeated runs of the same source–target pair are averaged first, the mean over pairs is reported with a 95% percentile bootstrap interval over pairs and DANN is compared with each other model using the paired Wilcoxon signed-rank test on the pair-level MAE.

#### 4.1.2. Evaluation Metrics

Calibration performance is evaluated with the Mean Absolute Error (MAE), the Root Mean Squared Error (RMSE), the coefficient of determination ($R^{2}$) and the mean bias $\bar{e}=\frac{1}{N}\sum_{n}\left({\hat{y}}_{n}-y_{n}\right)$, where a negative bias indicates underestimation of the reference. All errors are in μg/m^3^. RMSE penalises large errors more strongly than MAE, and the bias separates a systematic offset from random error. $R^{2}$ is computed against the mean of the evaluated reference series; it becomes strongly negative when that series has little variance, which is common for the short target records used here, so it is reported as an indicator of that situation and not as a ranking criterion. For PM_10_ and PM_2.5_, the uncalibrated sensor output is already in μg/m^3^ and is reported as a “Raw” baseline; the O_3_ and NO_2_ outputs are in mV and have no Raw baseline.

Beyond prediction accuracy, an important objective of this work is to assess the reproducibility of calibration models across different sensing devices and deployment locations. A model trained on a *source* device is therefore evaluated on an independent *target* device operating under the same or different environmental conditions.

To quantify calibration transferability, the Calibration Transfer Ratio (CTR) is defined as

$$CTR=\frac{MAE_{source}}{MAE_{target}}.$$

A value close to one indicates that calibration performance is preserved after transfer, while lower values indicate reduced transferability. A value closer to zero indicates model degradation caused by device-specific characteristics or changes in the deployment environment. A value greater than one implies the model performs better in the target device, which is rare but possible.

CTR is a ratio of two errors and carries no information about the absolute error: a model with a large source error and an equally large target error obtains CTR≈1, and a model with a very low source error can obtain a low CTR while still having a small target error. CTR is therefore always reported together with the absolute target-domain MAE and, where available, the Raw baseline (Table A13). Three evaluation scenarios are considered:

- Source domain evaluation (train and test on one device);
- Intra-location evaluation (different device within the same environment);
- Inter-location evaluation (different device deployed in a different geographical location).

These experiments provide insight into both calibration accuracy and model transferability.

#### 4.1.3. Domain Adaptation

Target domain evaluation often suffers from distribution shifts caused by differences in sensor manufacturing, installation conditions and local environmental characteristics. To improve model transferability, Domain Adversarial Neural Networks (DANNs) are investigated as a domain adaptation technique.

DANN extends conventional supervised learning by jointly optimising two competing objectives. A label predictor minimises the calibration error on the source domain, while a domain classifier, connected through a Gradient Reversal Layer (GRL), encourages the extracted features to become invariant to the deployment location [30]. Consequently, the learned representation retains information relevant for calibration while suppressing domain-specific characteristics.

The DANN model is trained using

- Labelled source-domain data containing sensor measurements and corresponding reference values;
- Unlabelled target-domain data containing only raw sensor measurements collected from other sensing devices or deployment locations.

This enables knowledge learned from a calibrated source device to be transferred to new deployments where reference measurements are unavailable.

Table 6 lists the DANN architecture and training configuration. The unlabelled target pool contains the feature vectors of all other devices, from both locations, and each device is its own domain class. Target reference values are never used. No other domain-adaptation method (for example CORAL, MMD-based adaptation or target fine-tuning) is evaluated. The learning rate and dropout rate are chosen by the lowest MAE on the training data of the source device.

### 4.2. Reference-Free Drift Detection Framework

Continuous reference measurements are unavailable for several modalities in the sensing platform, including CO_2_, CO, VOC, Lux, UV-A/B/C, PM_1_, temperature, humidity and pressure. Their health is therefore monitored using a reference-free framework that models the expected behaviour of each target sensor from the remaining measurements. Deviations from this learned relationship are analysed to identify changes consistent with sensor drift. The framework detects a loss of cross-sensor consistency; it does not identify which sensor is faulty.

**Figure 5:**
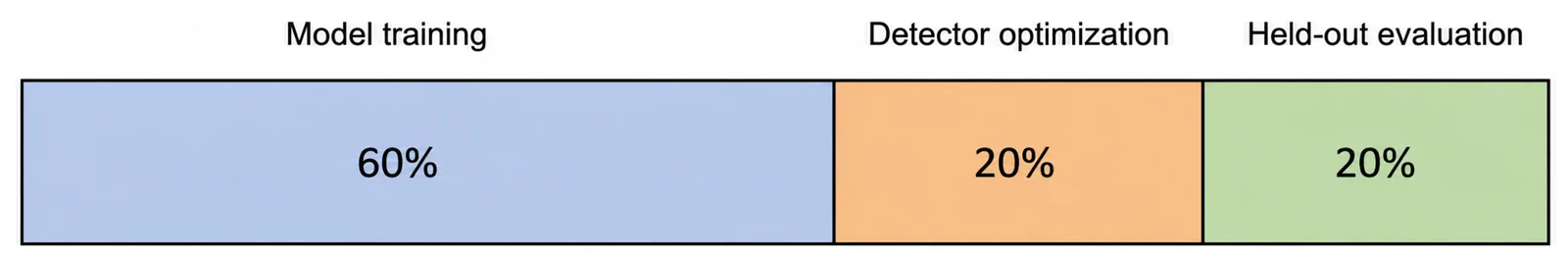
Data allocation for each stage of the proposed drift detection framework.

As illustrated in Figure 5, the framework consists of three stages: model training, detector optimisation and held-out evaluation. The available data for each device is divided into three partitions containing 60% for regression-model training, 20% for detector optimisation and 20% for final evaluation. The evaluation partition is one contiguous block starting at 40% of the record, the detector-optimisation partition is the final 20% of the record and the regression model is trained on the remaining data; the partitions are disjoint, and the standardisation parameters, detector parameters and ensemble rule are fitted on the detector-optimisation partition only.

#### 4.2.1. Model Training

For each target sensor $s_{i}$, an independent regression model is trained using healthy measurements from the remaining sensing modalities. The target measurement is excluded from the input feature vector, giving

$${\hat{s}}_{i}\left(t\right)=f_{i}\left(s_{1}\left(t\right),\ldots ,s_{i-1}\left(t\right),s_{i+1}\left(t\right),\ldots ,s_{n}\left(t\right)\right),$$

where $f_{i}\left(\cdot \right)$ denotes the selected regression model and ${\hat{s}}_{i}\left(t\right)$ is the estimated value of the target sensor at time *t*. The input vector consists of all other sensing channels of the device, and the regression model is a Random Forest with 100 trees and a maximum depth of 24.

The prediction residual is defined as

$$r_{i}\left(t\right)=s_{i}\left(t\right)-{\hat{s}}_{i}\left(t\right).$$

The residual represents the consistency of the target measurement with the cross-sensor relationships learned during healthy operation. When these relationships remain unchanged, the residual is expected to remain close to its healthy distribution. A persistent change in the residual indicates that the target measurement is no longer consistent with the remaining sensing modalities.

Residual magnitudes vary considerably between sensors because of differences in measurement units, operating ranges, measurement noise and regression-model accuracy. The residuals are therefore standardised before being analysed by the statistical tests.

The regression model is first applied to the healthy detector-optimisation partition. The resulting residual mean and standard deviation are calculated as

$$\mu_{i}=\frac{1}{N}\sum_{t=1}^{N}r_{i}\left(t\right),$$

and

$$\sigma_{i}=\sqrt{\frac{1}{N}\sum_{t=1}^{N}{\left(r_{i}\left(t\right)-\mu_{i}\right)}^{2}},$$

where *N* is the number of healthy samples in the detector-optimisation partition. Each residual is then transformed into a standardised residual,

$$z_{i}\left(t\right)=\frac{r_{i}\left(t\right)-\mu_{i}}{\sigma_{i}}.$$

The normalisation parameters $\mu_{i}$ and $\sigma_{i}$ are stored together with the regression model and remain fixed during detector optimisation and evaluation. This ensures that all residuals are assessed relative to the same healthy baseline.

#### 4.2.2. Detector Optimisation

**Figure 6:**
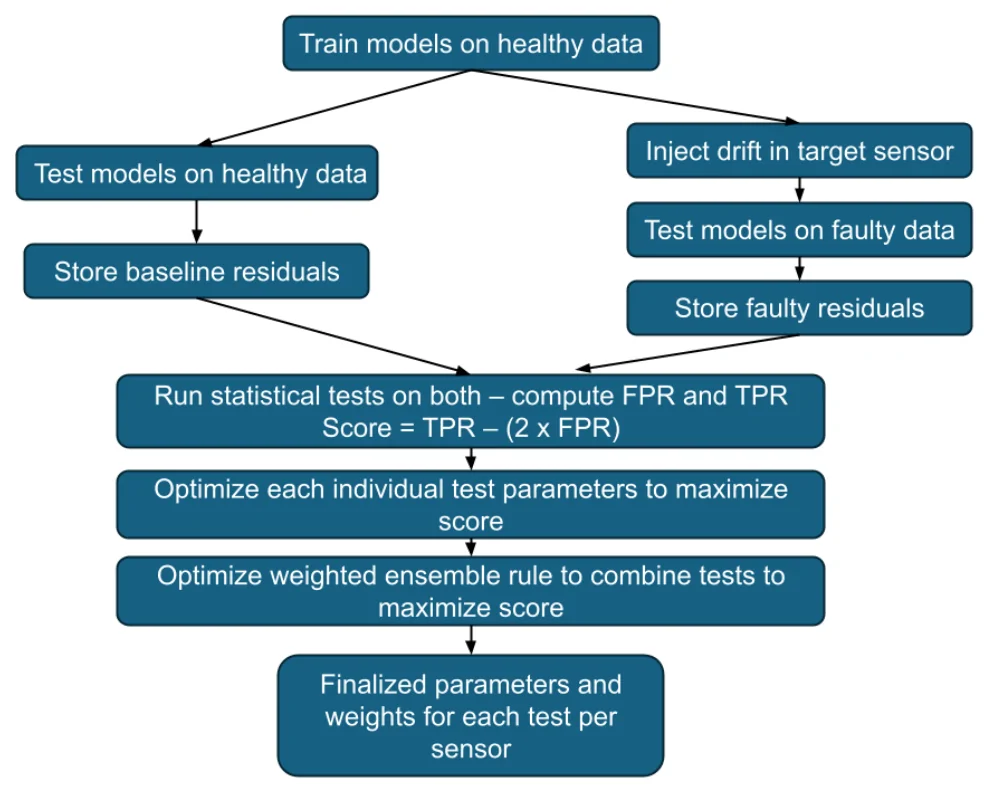
Detailed detector-optimisation procedure. Healthy and fault-injected residuals are used to optimise the individual statistical tests and their weighted ensemble for each target sensor.

Figure 6 presents the complete detector-optimisation procedure that is repeated for each target sensor. The trained regression model is first evaluated on unchanged healthy data to obtain the baseline residual sequence. Synthetic drift is then introduced into the target sensor, and the same model is applied to the resulting faulty sequence. The healthy and faulty residuals are analysed using the statistical tests defined in Section 3.5.3.

The metrics used to evaluate test accuracy are the true positive rate (TPR) and the false positive rate (FPR). The goal of the detector optimisation stage is to find the best parameters to maximise TPR and minimise FPR. The parameters of the individual tests are optimised first, followed by optimisation of the weighted ensemble decision rule.

**Synthetic drift injection.**

Long-term labelled examples of sensor drift are unavailable for most sensing modalities. Synthetic gradual drift is therefore introduced into the detector-optimisation data to produce a controlled sequence of faults. Drift is injected into one target sensor at a time while the remaining sensor measurements are left unchanged. This is the only fault model evaluated: abrupt offsets, gain errors, increased noise, stuck-at and intermittent faults, and simultaneous faults in several sensors are outside the scope of the reported experiments. The induced fault is represented as a linearly increasing additive bias,

$$e_{i}\left(t\right)=\left\{\begin{array}{cc} 0, & t<t_{0},\\ d_{i}\frac{t-t_{0}}{t_{N}-t_{0}}, & t_{0}\leq t\leq t_{N}, \end{array}\right.$$

where $t_{0}$ denotes the drift onset, $t_{N}$ is the final sample in the evaluation sequence and $d_{i}$ is the total drift magnitude applied to sensor *i*. The injected error, therefore, increases linearly from zero at $t_{0}$ to $d_{i}$ at $t_{N}$. The resulting faulty measurement is

$$s_{i}^{\prime }\left(t_{n}\right)=s_{i}\left(t_{n}\right)+e_{i}\left(t_{n}\right).$$

The drift magnitude is determined independently for each sensor and device from the range of its healthy evaluation data. Specifically,

$$d_{i}=0.25\left(Q_{0.9999}\left(s_{i}\right)-Q_{0.0001}\left(s_{i}\right)\right),$$

where $Q_{p}\left(s_{i}\right)$ denotes the *p*-quantile of sensor *i* in the evaluation set. Thus, the maximum injected drift corresponds to 25% of the range for that sensor. Because the two extreme quantiles are estimated from the outermost samples of the evaluation partition, this range is sensitive to single outliers.

**Figure 7:**
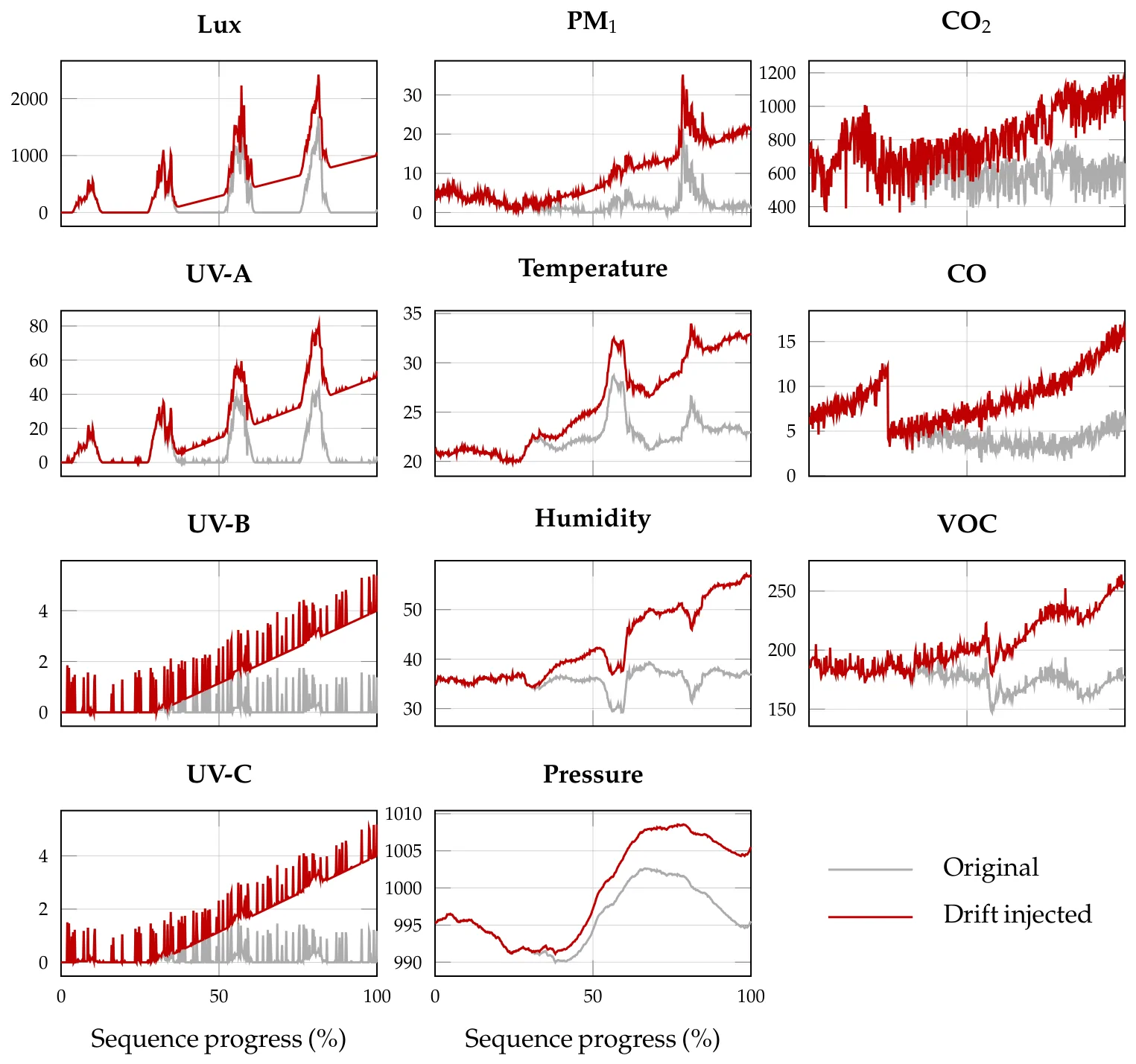
Synthetic gradual drift injected independently into the evaluated sensor measurements. The original measurements are shown together with the corresponding fault-injected sequences.

Drift begins after 30% of the detector-optimisation partition, leaving an initial healthy interval before the fault onset. Figure 7 illustrates the injected gradual drift for the evaluated sensing modalities.

The trained regression model is applied to the fault-injected sequence without retraining. The resulting faulty residuals are standardised using the same healthy baseline parameters $\mu_{i}$ and $\sigma_{i}$,

$$z_{i}^{\mathrm{faulty}}\left(t\right)=\frac{s_{i}^{\prime }\left(t\right)-{\hat{s}}_{i}\left(t\right)-\mu_{i}}{\sigma_{i}}.$$

This produces a healthy standardised residual sequence and a corresponding fault-injected sequence for detector optimisation.

**Statistical-test optimisation.**

The Z-score, CUSUM, EWMA and rolling-mean detectors, along with their parameter ranges, are defined in Section 3.5.3. Each statistical test is applied independently to the healthy and fault-injected residual sequences. For the statistical test *k*, the binary output is denoted by

$$d_{i,k}\left(t\right)\in \left\{0,1\right\},$$

where $d_{i,k}\left(t\right)=1$ indicates that the residual of target sensor $s_{i}$ is considered abnormal.

The parameter combinations of each test are evaluated separately. A persistence condition is subsequently applied, requiring that an alarm remain active for at least 15 consecutive samples. This suppresses isolated detections caused by short residual fluctuations. The value of 15 samples is a design choice; it corresponds to about 90 s during charging and about 37 min during battery operation (Table 2).

Each candidate configuration is assessed using

Score=TPR−2FPR,

where TPR is calculated over the post-fault interval, and FPR represents alarms generated under healthy operation. Configurations with an FPR exceeding 0.30 incur an additional penalty. A factor of two places greater emphasis on reducing false alarms, since a detector with high sensitivity but frequent alarms would have limited practical value. The score is a cost-weighted variant of Youden’s index J=TPR−FPR [45] in which a false alarm counts twice as much as a missed detection; the weight of two and the FPR limit of 0.30 are design choices. The penalty is a constant of −100 for configurations whose FPR exceeds 0.30, so such configurations are chosen only if no configuration satisfies the limit.

**Weighted ensemble optimisation.** After the parameters of the individual tests have been selected, their binary outputs are combined through a weighted decision rule. Each test is assigned an integer weight,
  $$w_{i,k}\in \left\{0,1,2\right\},$$
  where a weight of zero excludes the test from the final decision, a weight of one gives it standard importance, and a weight of two gives it greater influence. The combined detection score is
  $$D_{i}\left(t\right)=\sum_{k=1}^{K}w_{i,k}d_{i,k}\left(t\right),$$
  where *K* is the number of statistical tests. An initial drift alarm is generated when
  $$D_{i}\left(t\right)\geq T_{i},$$
  where $T_{i}$ is the ensemble decision threshold for target sensor $s_{i}$.

All non-zero combinations of the statistical-test weights are evaluated together with the candidate decision thresholds. The same persistence requirement of 15 consecutive samples is applied to the combined output. Candidate ensemble configurations are ranked using the TPR–FPR objective defined above. If multiple configurations obtain the same score, preference is given to higher TPR, lower FPR, and shorter detection delay. Detection delay is the number of samples between fault onset and the first persistent alarm in the evaluation partition.

**Figure 8:**
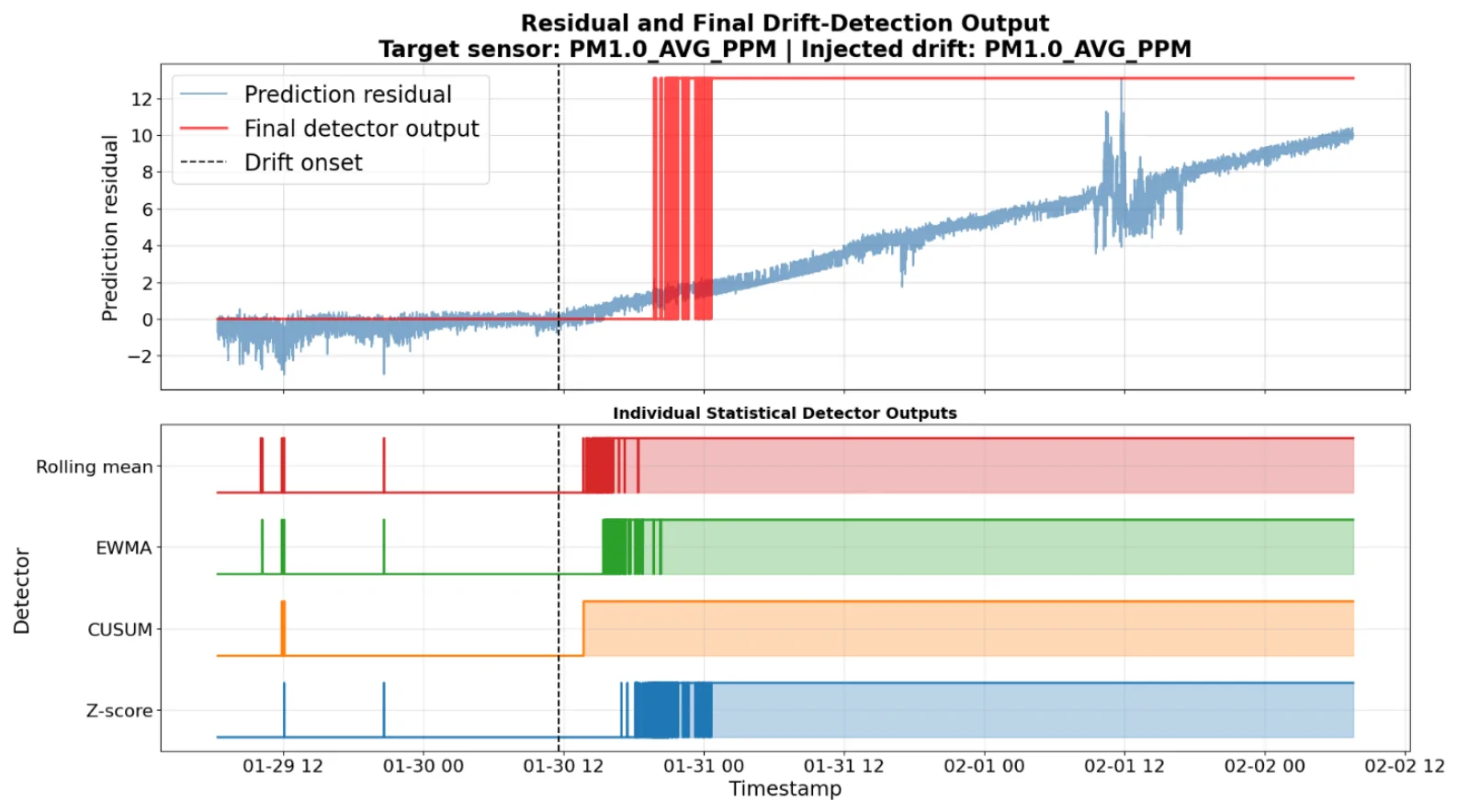
Combination of the individual statistical test outputs using the optimised weights and decision threshold. The final drift flag is generated from the weighted ensemble and persistence rule.

Figure 8 illustrates how the individual statistical test outputs are combined to produce the final persistent fault flag.

The optimisation is repeated independently for every target sensor. The resulting detector contains the trained regression model, its input feature list, the residual-normalisation parameters, the optimised parameters for each statistical test, the ensemble weights, the decision threshold and the persistence requirement. These components remain fixed when the detector is applied to the held-out evaluation data. The evaluation procedure and resulting TPR, FPR and detection-delay measurements are presented in Section 5.

## 5. Results

### 5.1. Sensor Calibration

The calibration framework was evaluated for the four pollutants with available reference measurements: O_3_, NO_2_, PM_2.5_ and PM_10_. Three evaluation scenarios were considered:

1. Source-domain evaluation, where a model was tested on the same device used for training;
2. Intra-location transfer, where the model was applied to another device at the same location;
3. Inter-location transfer, where the model was applied to a device at a different location.

The experiments were repeated with each available device acting as the source device. The bar plots aggregate the results with the device-balanced procedure of Section 4.1.1: bar height is the mean over source devices (source scenario) or over source–target pairs (transfer scenarios), after repeated runs of a pair have been averaged; error bars are 95% percentile-bootstrap intervals over those units, and each dot is one device or device pair. Exact values, RMSE, $R^{2}$, bias and Wilcoxon tests are given in Appendix C. With only 5 source devices and 6–12 transfer pairs, most intervals are wide and few differences between models are statistically distinguishable.

Calibration accuracy was evaluated using Mean Absolute Error (MAE) (RMSE, $R^{2}$ and CTR in the Appendix C). Transferability was evaluated using the Calibration Transfer Ratio (CTR), defined in Section 4.1.2. Since CTR is calculated as the ratio of source-domain to target-domain MAE, values close to 1 indicate that most of the source-domain performance was preserved after transfer.

#### 5.1.1. Source-Domain Performance

**Figure 9:**
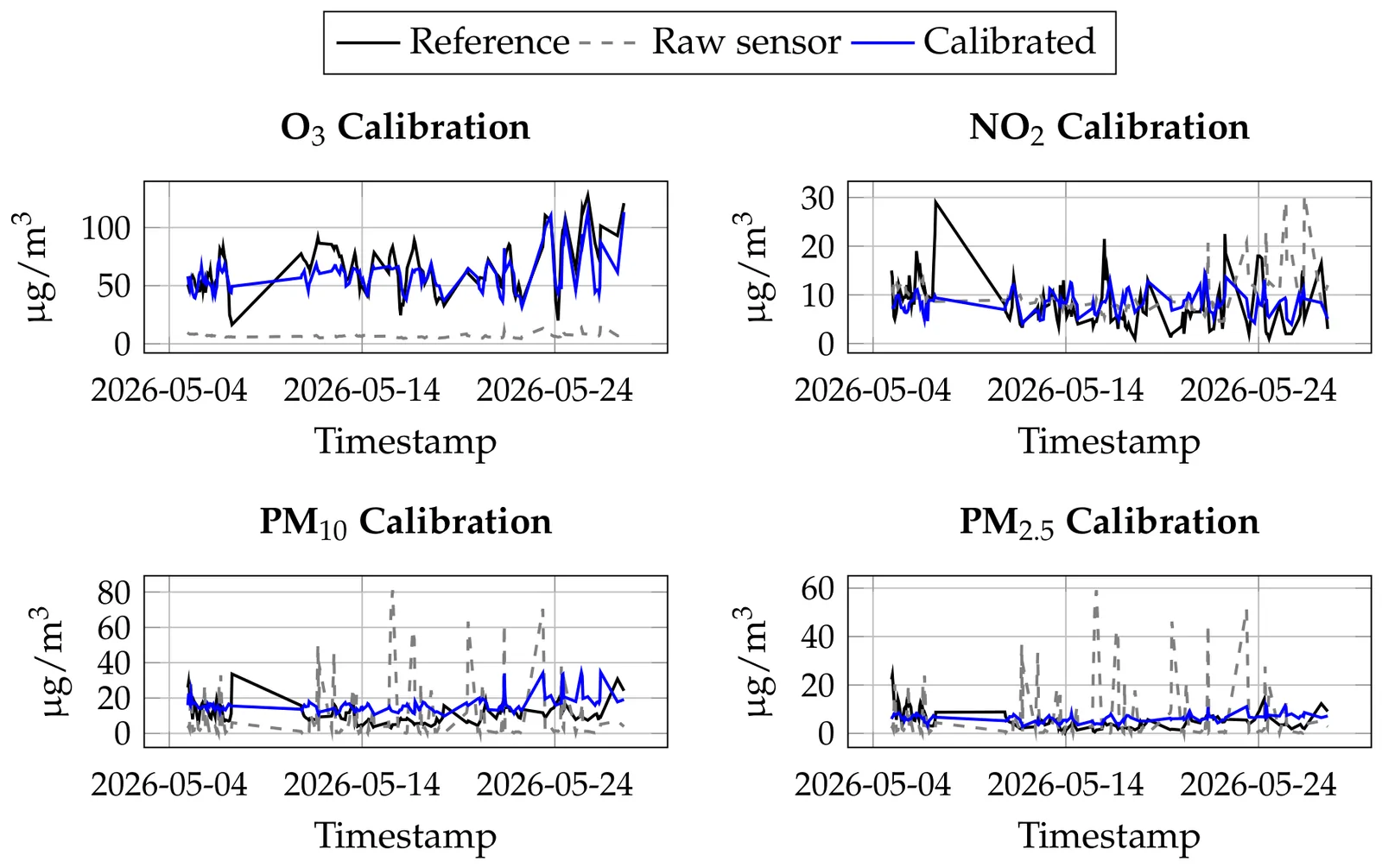
Comparison of raw, calibrated and reference measurements for O_3_, NO_2_, PM_10_ and PM_2.5_ (concentrations in μg/m^3^; the raw O_3_ and NO_2_ curves are sensor voltages in mV plotted on the same axis for shape comparison only).

**Figure 10:**
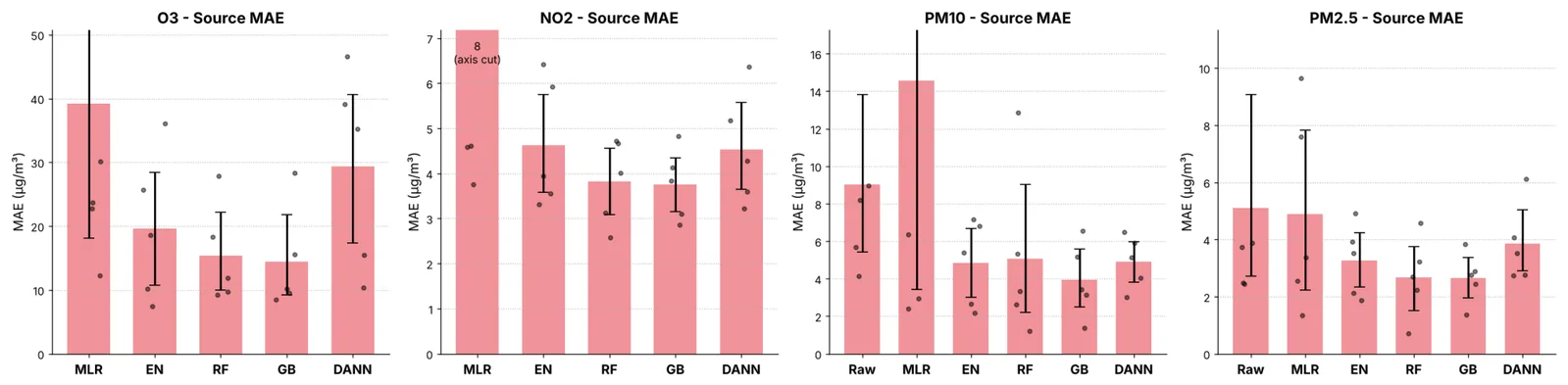
Device-balanced source-domain MAE in μg/m^3^. Bars: mean over the five source devices; error bars: 95% bootstrap interval over devices; dots: individual devices. The MLR bars are truncated where marked. No Raw bar is shown for O_3_ and NO_2_ because the raw output is in mV.

Figure 9 compares the raw sensor measurements, calibrated predictions and reference measurements for a representative source device. Only the best-performing model for each pollutant is shown for clarity. The comparison across all models and source devices is presented in Figure 10.

The calibrated predictions generally follow the reference measurements more closely than the raw sensor outputs. The improvement is clearer for O_3_ and NO_2_ than for PM_2.5_ and PM_10_. The raw PM measurements were already relatively close to the reference values, leaving less room for improvement through calibration.

RF and GB give the lowest source-domain MAE for O_3_ (15.4 and 14.4 μg/m^3^) and NO_2_ (3.8 and 3.8 μg/m^3^) and for PM_2.5_ (2.7 and 2.7 μg/m^3^, against 5.1 for the raw sensor). For PM_10_, GB is best (3.9 μg/m^3^), and RF (5.1) is no better than EN (4.8) or DANN (4.9); all of them improve on the raw sensor (9.0). MLR is the weakest model for every pollutant and, for PM_10_ and PM_2.5_, is no better than, or worse than, the raw output. EN is clearly better than MLR and close to the tree models for NO_2_ and PM. DANN is competitive for NO_2_ and PM (4.5, 4.9 and 3.8 μg/m^3^) but clearly worse than RF and GB for O_3_ (29.3 μg/m^3^).

The intervals of RF, GB, EN and DANN overlap for NO_2_, PM_10_ and PM_2.5_; a Wilcoxon test over the five source devices finds no significant difference between DANN and any other model (smallest p=0.0625, which is the lowest value attainable with five devices). The ranking among the leading models is therefore indicative only. Absolute fit is also modest: the pooled $R^{2}$ on the held-out source blocks is close to or below zero for several model and pollutant combinations (Appendix C Table A13) because the held-out blocks are short and have small variance.

The weak O_3_ result of DANN is accompanied by a large negative bias: the mean source-test bias is −28 μg/m^3^ (per device −55, −34, −15, −5 and −34 μg/m^3^), i.e., the network systematically underestimates ozone. The error is therefore mostly a systematic offset and not noise. A plausible cause is that the adversarial term, together with a model selected on training MAE after a fixed 100 epochs, moves the shared representation away from the device-specific O_3_ offset that the other models exploit; this explanation was not tested.

#### 5.1.2. Intra-Location Transfer

**Figure 11:**
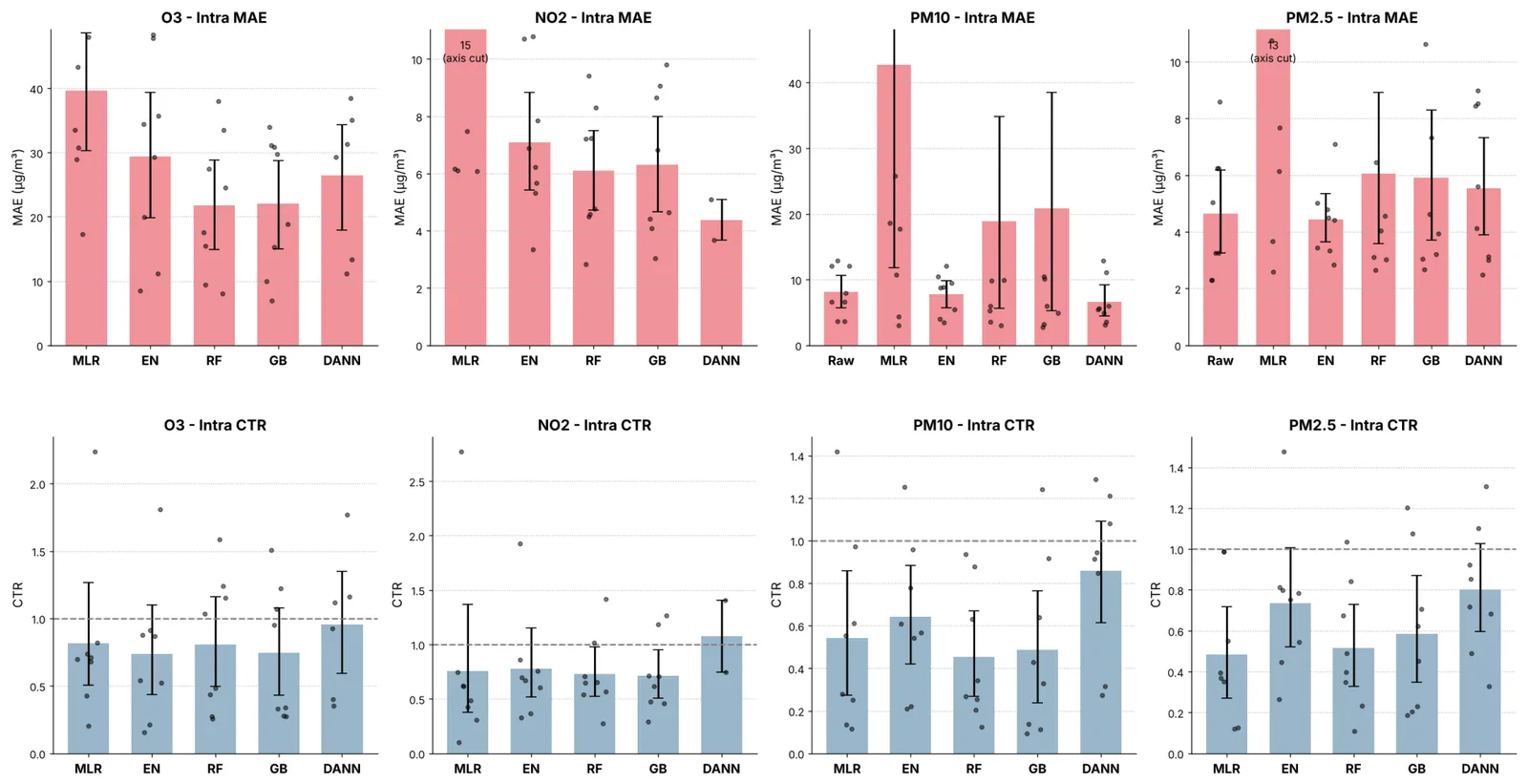
Device-balanced intra-location transfer: target-domain MAE in μg/m^3^ (**upper**) and CTR (**lower**) with 95% bootstrap intervals over source–target pairs; dots are individual pairs. Pairs: 8 (6 for DANN on O_3_ and 2 for DANN on NO_2_, where fewer DANN runs were available).

The intra-location evaluation considers transfer between different devices deployed at the same location. Since the devices experience similar environmental conditions, the primary source of domain shift is variation among individual sensor units. Figure 11 shows the aggregate MAE and CTR across all valid intra-location source–target combinations.

DANN has the highest mean CTR for all four pollutants (0.95 for O_3_, 1.07 for NO_2_, 0.86 for PM_10_ and 0.80 for PM_2.5_), followed by EN (0.74, 0.78, 0.64 and 0.73); RF and GB are between 0.45 and 0.81, and MLR between 0.49 and 0.81. However, the intervals overlap throughout (for example, O_3_ DANN 0.59–1.35 against RF 0.50–1.16), individual pairs range from about 0.1 to above 2, and no paired difference between DANN and another model is significant (smallest p=0.0625). Part of DANN’s higher CTR comes from its larger source error (CTR is source error divided by target error), and the DANN means rest on fewer pairs for O_3_ and NO_2_.

Absolute errors give a different ordering for some pollutants. For O_3_, RF and GB remain best (21.7 and 22.1 μg/m^3^) and DANN is next (26.4). For NO_2_, DANN is best (4.4 μg/m^3^, from two pairs), ahead of RF, GB and EN (6.1, 6.3, 7.1). For PM_10_, DANN (6.6) and EN (7.8) improve slightly on the raw sensor (8.2), while RF and GB do not (18.9 and 20.9, driven by a few pairs with very large errors) and MLR is far worse (42.7). For PM_2.5_, no model improves on the raw sensor (4.6): EN gives 4.4 and DANN 5.5. A high CTR therefore did not imply a low target error, and for PM, the transferred calibrations did not beat the uncalibrated sensor.

#### 5.1.3. Inter-Location Transfer

The inter-location evaluation considers transfer between devices deployed at different locations. This introduces both device-to-device variation and differences in pollutant distributions and environmental conditions.

**Figure 12:**
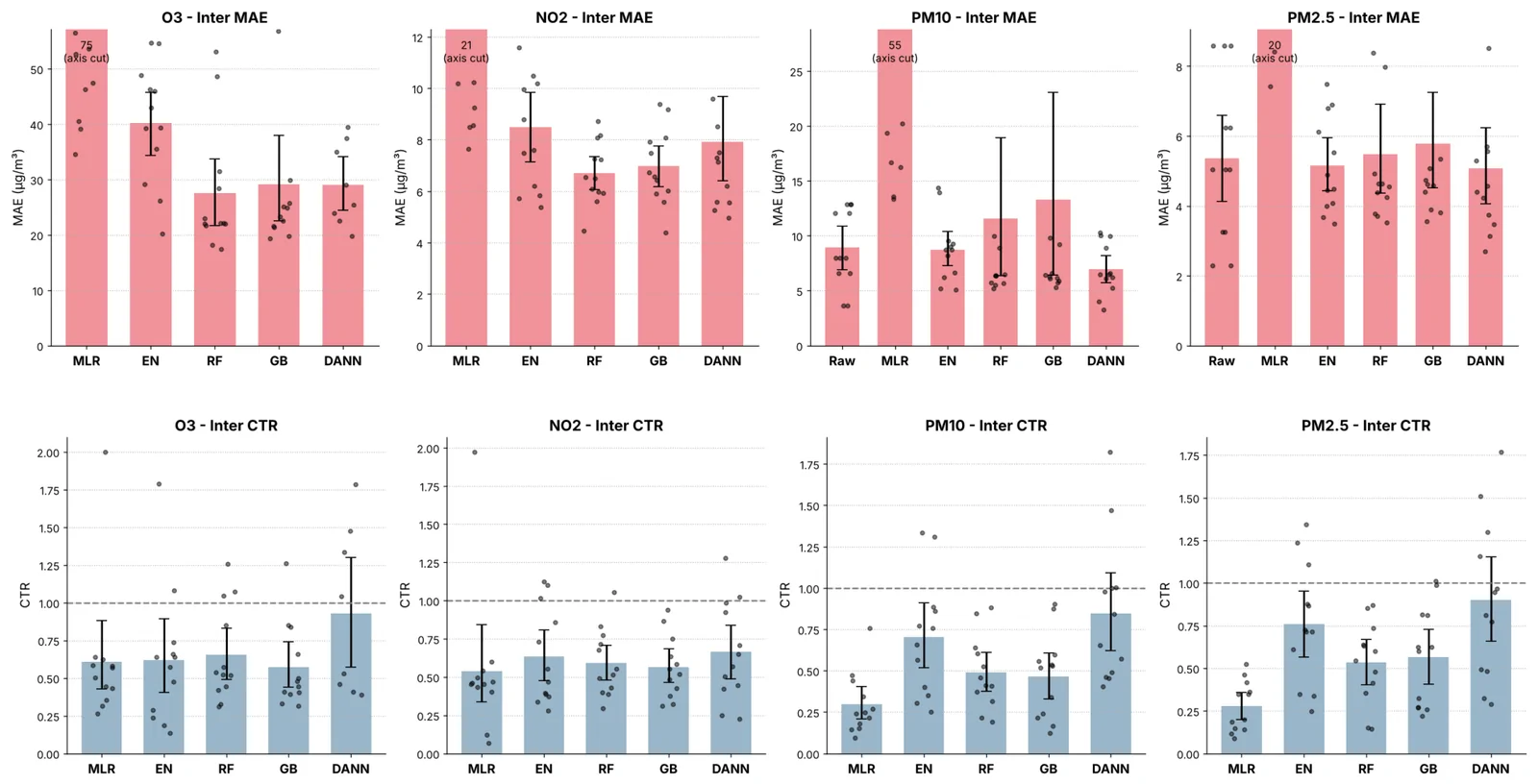
Device -balanced inter-location transfer: target-domain MAE in μg/m^3^ (**upper**) and CTR (**lower**) with 95% bootstrap intervals over source–target pairs; dots are individual pairs (12 pairs; 8 for DANN on O_3_).

Figure 12 shows the aggregate MAE and CTR across all valid inter-location source–target combinations.

CTR values are lower than for intra-location transfer for NO_2_ and PM and similar for O_3_ with DANN. DANN again has the highest mean CTR (O_3_ 0.93, NO_2_ 0.67, PM_10_ 0.85 and PM_2.5_ 0.90); EN follows for NO_2_, PM_10_ and PM_2.5_ (0.64–0.76); and RF, GB and MLR lie between 0.28 and 0.66. As in the intra-location case, the intervals overlap and none of the paired differences is significant (smallest p=0.15).

In absolute terms, RF has the lowest O_3_ error (27.5 μg/m^3^), with GB and DANN close behind (about 29), and EN is clearly worse (40.3). For NO_2_, RF and GB (6.7, 7.0) are slightly better than DANN (7.9) and EN (8.5). For PM_10_, DANN (7.0) is better than the raw sensor (8.9) and EN (8.7) is on par with it, whereas RF and GB (11.6, 13.3) are worse than the raw sensor and MLR is far worse (55). For PM_2.5_, all models except MLR fall within about ±0.5 μg/m^3^ of the raw sensor (5.4), with DANN (5.1) and EN (5.2) marginally ahead. Under inter-location transfer, therefore, the tree ensembles do not retain an advantage for PM, and DANN’s advantage in CTR is mostly a consistency advantage rather than a demonstrated accuracy advantage. Because the Halle PM reference comes from a station about 11 km away (Section 3.2), part of the PM error in transfers involving Halle is reference-representativeness error and cannot be attributed to the sensor model.

#### 5.1.4. Overall Calibration Performance

Taken together, the results support three statements. (i) RF and GB are the most accurate models on the source device for the gases and for PM_2.5_, and they are the most accurate for O_3_ in both transfer settings and for NO_2_ between locations; this supports using them when a calibration can be fitted for each deployment. (ii) DANN retains the largest fraction of its source error under transfer (highest mean CTR in all eight transfer settings) and gives the lowest target error for PM_10_ in both transfer settings, but its source accuracy for O_3_ is poor and its advantages over the other models are not statistically significant with the available device pairs, so we do not claim that it is generally the best approach. (iii) For PM, transferred calibrations (RF and GB in particular) can be worse than the uncalibrated sensor, so a calibration should be checked against a local reference before it is used on a new device. MLR performs poorly in every scenario, and EN transfers acceptably between devices but degrades across locations for O_3_.

All conclusions are conditional on five devices, two locations, 9 to 68 days of data per device, reference stations several kilometres from the devices and the evaluation protocol of Section 4.1.1.

### 5.2. Drift Detection Evaluation

The proposed framework was evaluated on all sensors without continuously available reference measurements, including PM_1_, CO_2_, VOC, Lux, UV-A/B/C, temperature, humidity and pressure. For each sensor, an independent regression model was trained using healthy data, after which synthetic gradual drift was injected into the target sensor while leaving all remaining sensors unchanged. The resulting residual sequences were analysed using the statistical monitoring framework described in Section 4.2.

#### 5.2.1. Residual Behaviour

**Figure 13:**
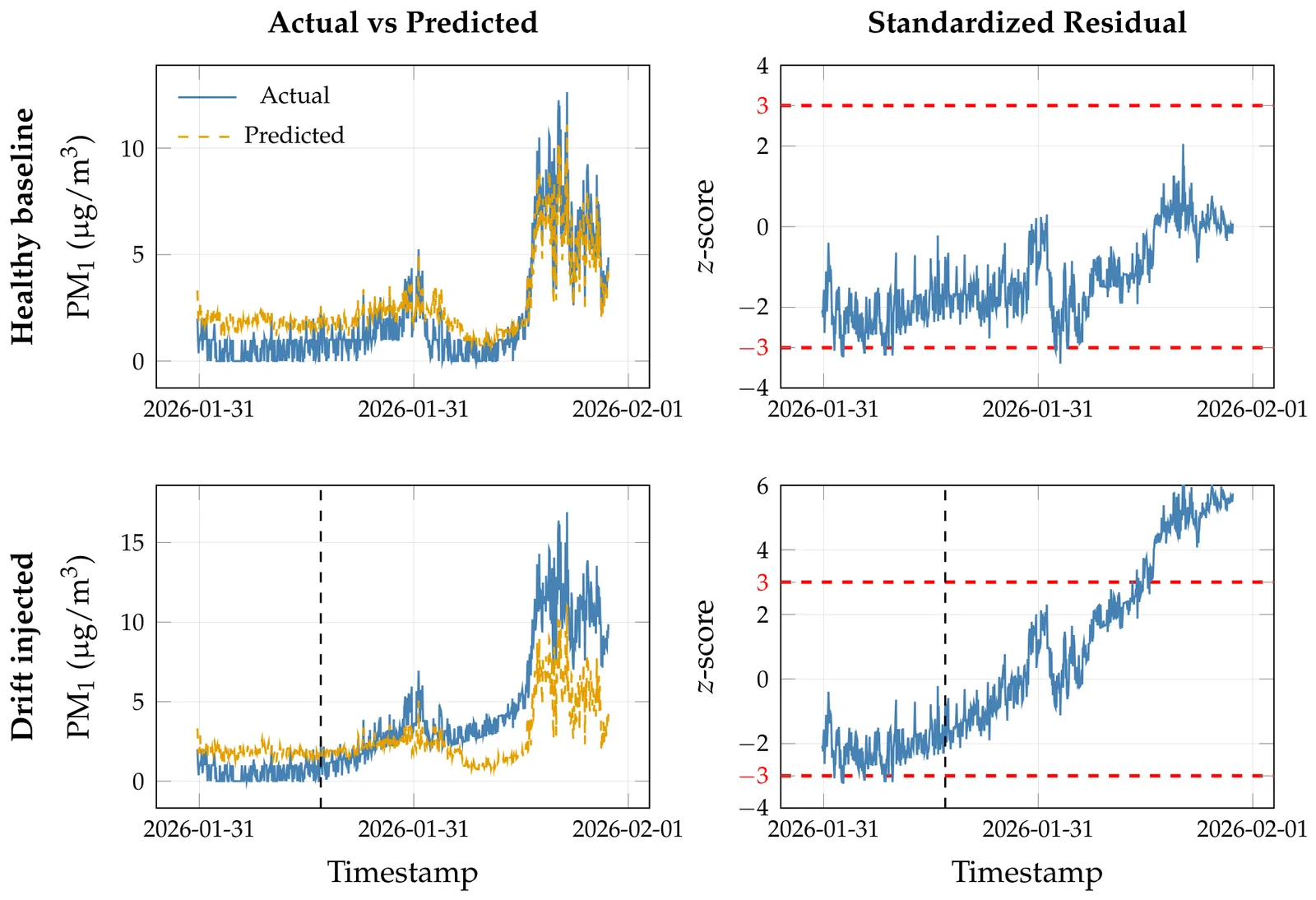
PM_1_ model behaviour under healthy operation and following synthetic drift injection. The left panels compare measured and predicted PM_1_ values, while the right panels show the corresponding standardised residuals. The dashed vertical line indicates the onset of drift.

Figure 13 illustrates representative residual sequences for the PM_1_ sensor under healthy and drifted operating conditions, respectively.

Under healthy conditions, residuals remain centred around zero with only small random fluctuations caused by modelling uncertainty and measurement noise. Consequently, the normalised residual sequence remains statistically stationary, allowing the statistical monitoring algorithms to maintain a low false alarm rate.

Following drift injection, the residual distribution progressively shifts away from zero as the disagreement between the measured and predicted sensor values increases. This persistent bias is effectively captured by statistical monitoring techniques, particularly CUSUM and EWMA, which are designed to detect slowly varying changes.

#### 5.2.2. Detection Performance

For every sensor, the optimised statistical ensemble was evaluated using both healthy and fault-injected datasets. Detection performance was quantified using the True Positive Rate (TPR), representing the proportion of injected fault samples correctly identified, and the False Positive Rate (FPR), representing the proportion of healthy measurements incorrectly classified as drifting.

**Figure 14:**
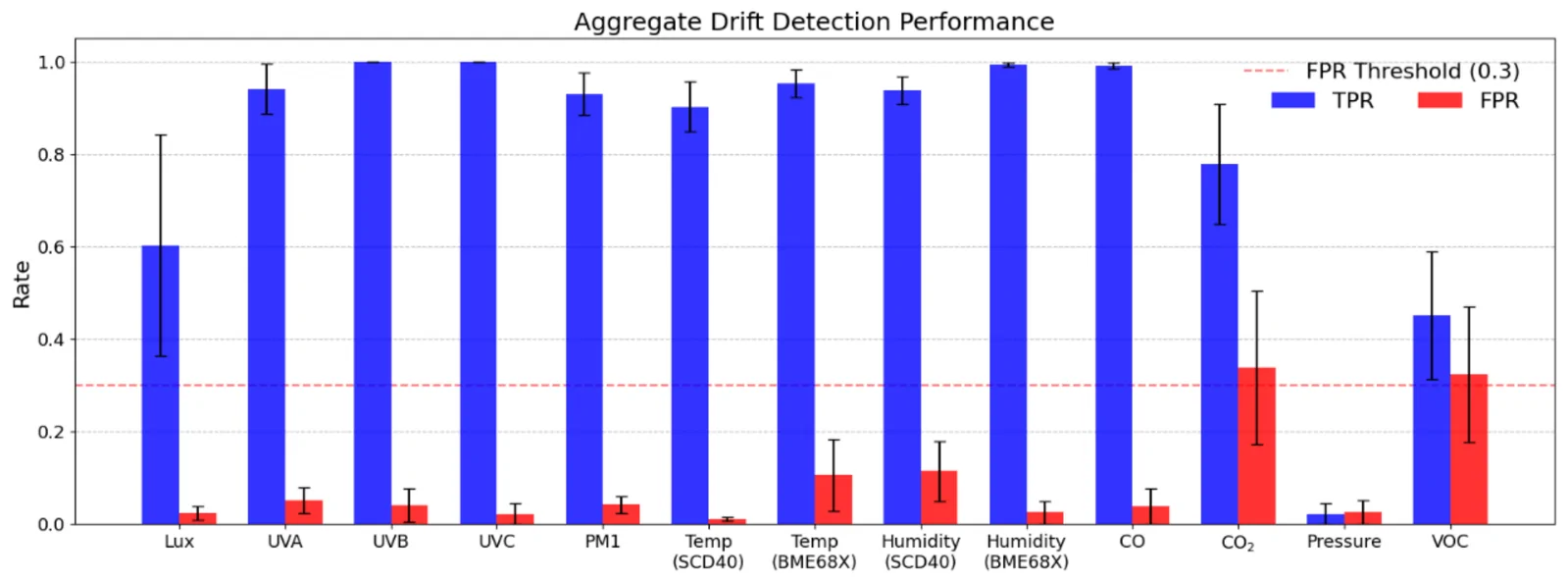
Aggregate drift-detection performance across the evaluated sensing modalities. Bars show the mean TPR and FPR across devices, while the error bars indicate device-to-device variability.

Figure 14 summarises the final detection performance across all evaluated sensors.

The highest detection performance is obtained for the UV channels, PM_1_, and the duplicated temperature and humidity measurements. These targets combine high TPR with comparatively low healthy-data FPR. CO also achieves useful detection performance despite having no single strong pairwise correlate, indicating that several weaker relationships can collectively provide sufficient predictive information.

CO_2_ produces more variable results between devices, while pressure and VOC are the least reliably detected targets. Unlike particulate or optical measurements, these variables possess weaker relationships with the remaining sensors, reducing the predictive accuracy of the regression models and increasing the baseline residual variance. As a result, distinguishing genuine sensor drift from normal modelling error becomes significantly more challenging.

**Figure 15:**
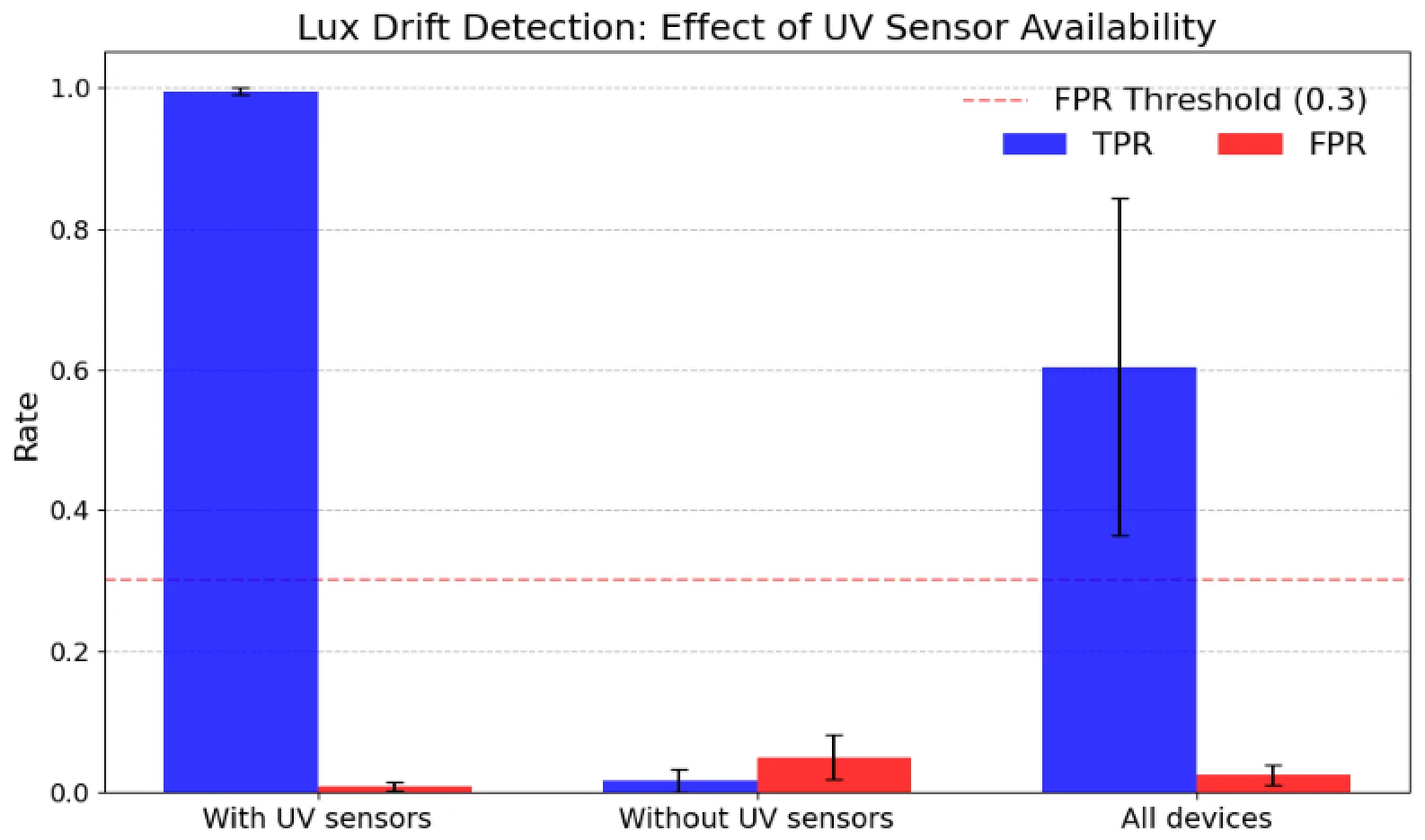
Effect of UV-sensor availability on Lux drift detection. Performance is shown separately for devices containing UV measurements, devices without UV measurements, and the complete device set.

Lux exhibits lower aggregate TPR and greater device-to-device variability than the UV channels. However, only three of the five evaluated devices contain the UV sensor. Figure 15, therefore, separates the Lux results for devices with UV measurements, devices without UV measurements and the complete device set.

Lux detection is substantially stronger on devices containing UV-A, UV-B and UV-C measurements. When these channels are unavailable, Lux must be estimated from weaker environmental and temporal relationships, and detection performance decreases sharply. The comparison demonstrates empirically that the detectability of a target differs according to the supporting modalities available in the device configuration.

Overall, the results demonstrate that reference-free drift detection is most effective when the target sensor exhibits strong physical or statistical relationships with other sensing modalities. Detection performance degrades as sensor redundancy decreases, highlighting the importance of cross-sensor information for autonomous health monitoring.

#### 5.2.3. Interpretation of the Detection Rates

Two points qualify the rates above. First, they were obtained against one injected fault model, a single linear additive drift reaching 25% of the evaluation-set range in one sensor at a time. They describe sensitivity to that model and are not evidence of detecting real sensor ageing. Second, the healthy-data false-positive rate is not uniformly low. The mean FPR is 0.34 for CO_2_ and 0.32 for VOC, and 6 of the 56 device–sensor combinations exceed the 0.30 bound used during tuning (Halle_2 CO_2_ 0.87; Ukkel_2 VOC 0.86, CO_2_ 0.53, BME680 temperature 0.41 and SCD40 humidity 0.33; Ukkel_1 VOC 0.38). For these cases, a high TPR is not meaningful because an alarm that is active for a third or more of the healthy samples also fires after the fault. Pressure has a mean TPR of 0.02, so the injected drift is essentially never detected, and Lux has a mean TPR of 0.60 with a range of 0 to 1 across devices. The FPR limit of 0.30 is therefore a feasibility bound for the optimisation and not an acceptable operating point. Practical monitoring would need a much stricter FPR target, which lowers the TPR and lengthens the delay, or aggregation of alarms over longer windows. Detection delay was used only as the last tie-breaker when ranking equally scored configurations and is not reported as a result.

## 6. Discussions

This study investigated calibration and reference-free drift detection in a multi-modal environmental sensing platform operating with incomplete reference coverage. The findings address the three research questions introduced in Section 1, within the limits of five devices, two locations and one injected-fault model.

For **RQ1**, no calibration model was optimal across all deployment scenarios. Random Forest and Gradient Boosting achieved the strongest source-domain calibration, indicating that nonlinear models were better able to capture the relationships between raw pollutant measurements, environmental variables and the remaining sensing modalities. Their advantage, however, decreased when the models were transferred to other devices and locations.

DANN retained a larger proportion of its source performance under transfer (highest mean CTR in all eight transfer settings) and gave the lowest PM_10_ target error in both transfer settings. These differences were not statistically significant with the available device pairs; DANN’s source accuracy for O_3_ was poor (large negative bias); and for PM_2.5_, no model clearly improved on the uncalibrated sensor after transfer. The evidence therefore does not establish DANN as generally superior; it indicates that DANN is a reasonable candidate when a model must be moved between devices or locations and some unlabelled target data exist, and that RF and GB are preferable when calibration can be performed separately for each deployment. Model selection should therefore depend on the intended deployment strategy. Transferability should nevertheless be assessed using both CTR and absolute target-domain error, since a high CTR does not necessarily imply accurate target predictions; this was observed here for PM_2.5_ and PM_10_, where transferred models with moderate CTR were sometimes no better than the raw sensor.

For **RQ2**, the results show that cross-sensor dependencies can support reference-free drift detection when the target measurement can be predicted reliably from the remaining modalities. Strong detection with low false-alarm rates was observed for UV, PM_1_, CO, temperature and humidity measurements, where substantial redundancy or correlation was available. Detection of Lux depended strongly on the device and on UV availability, and CO_2_ and VOC reached useful TPR only at false-positive rates of about 0.3 or more, which would not be acceptable in practice. Pressure and VOC were more difficult to monitor because their healthy residuals were less stable and their behaviour was less accurately reconstructed than that of the other sensors.

The framework should therefore be interpreted as detecting changes in learned cross-sensor consistency rather than directly proving that a particular sensor has drifted. This distinction matters for calibration as well: the calibration models take other modalities as inputs, so a drifting predictor would corrupt calibrated outputs without any change in the target sensor, and the present experiments do not quantify that effect. Likewise, calibration errors were not propagated into the drift monitor, and the two parts of the work are evaluated separately. A fault in one measurement may affect the residuals of several correlated models, thereby improving sensitivity to inconsistency but complicating the fault isolation. This is particularly important when related outputs originate from the same sensing module.

For **RQ3**, intra-location transfer generally preserved more calibration performance than inter-location transfer, with most intra-location CTR values remaining between 0.8 and 1.0. This suggests that device-to-device variability within a deployment area was smaller than the combined device and environmental shift introduced by transfers between locations. The magnitude of this inter-location shift differed between pollutants. O_3_ retained the highest CTR values across all models, whereas NO_2_, PM_2.5_ and PM_10_ showed greater degradation. One possible explanation is that O_3_ exhibited less spatial variation between the two deployment locations, while NO_2_ and particulate matter were more strongly influenced by local sources. At the municipality scale, Halle combines a small urban centre and surrounding open space with the influence of the major highways, whereas Ukkel is a greener municipality embedded within the Brussels urban environment. Differences in land use and road-traffic influence could therefore have contributed to the observed inter-location degradation. However, because the exact device coordinates and corresponding local traffic and land-use data were unavailable, this remains a contextual interpretation rather than a demonstrated causal explanation. A second, more mundane explanation is also open: the Halle PM reference comes from a station about 11 km away, so part of the inter-location PM degradation may reflect reference representativeness instead of local-source differences.

The available reference measurements only permit domain-shift analysis for O_3_, NO_2_, PM_2.5_ and PM_10_. The behaviour of the remaining sensing modalities under geographical domain shift was not evaluated. Their analysis was limited to cross-sensor predictability and controlled synthetic drift.

Overall, the findings support a deployment-aware reliability strategy. Calibration models should be selected according to the expected degree of domain shift, while residual-based monitoring should be applied primarily to modalities for which stable cross-sensor relationships can be demonstrated.

## 7. Limitations

The calibration study uses five devices deployed across two locations. This provides multiple source–target combinations, but it remains a limited sample of the variability expected in a large-scale European deployment. Devices, locations and observation periods are not equally represented, and the duration and number of reference-aligned measurements differ between datasets. Although the aggregation procedure prevents repeated source results from receiving additional weight, the target-domain estimates are still based on the available ordered device pairs rather than on a balanced experimental design.

The transfer results may therefore be influenced by the particular devices and environmental conditions present in Halle and Ukkel. A model that performs well between these domains is not guaranteed to generalise to a new sensor batch, climate, installation configuration or pollutant distribution. Independent validation using devices and locations excluded from all model development would provide a stronger test of geographical generalisation. Several data channels (UV and CO) are missing on some devices, the Halle and Ukkel records cover different periods and seasons, the month-of-year feature may act as a deployment-period identifier for the short Ukkel records, reference stations are several kilometres from the devices (about 11 km for Halle PM), and the evaluation protocol differs between Halle (month-wise held-out blocks) and Ukkel (final block). With a handful of device pairs, confidence intervals are wide and the model comparisons are exploratory. The contribution of the multi-modal inputs relative to target-only, or temperature- and humidity-only inputs; of each detector relative to the ensemble; and of the adversarial term relative to other adaptation methods was not evaluated separately.

The drift-detection evaluation uses synthetic gradual drift rather than labelled physical sensor failures. For each target and device, the injected drift magnitude is defined as 25% of the evaluation-set range (computed from extreme quantiles, which are sensitive to outliers). This produces a controlled and comparable experiment but does not establish how the detector will respond to the magnitude, shape or timing of drift arising from real sensor ageing. The reported TPR is performance against this injected fault model only. The false-positive rate of up to 0.3 allowed during tuning is not acceptable for practical monitoring, and several detectors exceeded it on held-out data. Persistence, score weighting and the FPR bound are design choices without a theoretical optimum. Detection delay, ROC curves and sensitivity to the drift magnitude were not evaluated; performance is reported at the selected operating point only. In the drift pipeline the native sampling rate was kept, so charging periods carry many more samples than battery periods.

Only one sensor is modified at a time while all predictor measurements remain unchanged. Practical deployments can contain simultaneous faults, common-mode environmental interference, communication errors, abrupt offsets, increased noise, scaling errors, intermittent measurements or failures affecting a complete sensing subsystem. These conditions may alter several residuals at once and could be more difficult to isolate than the evaluated single-target case.

The method assumes that the relationships learned during the healthy training period remain sufficiently stable during monitoring. A new environmental regime can change these relationships even when every sensor is physically healthy, potentially increasing the false-alarm rate. Conversely, correlated drift affecting both a target and its strongest predictor could preserve their relationship and remain undetected.

Most importantly, the framework detects inconsistency but does not isolate its physical cause. A target residual can change because the target is drifting, because one of its predictors is faulty or because the environment has moved outside the learned operating domain. Independent diagnostic evidence is required before removing, replacing or recalibrating a sensor in response to an alarm. Calibration models also use other modalities as predictors, which may drift themselves, and calibration and drift detection are evaluated separately, so the effect of calibration error on monitoring and of monitoring alarms on calibration was not studied.

**On-device cost.** Both parts were run offline in *Python*-3.12.10 and no timing, memory or energy measurement was made on the ESP32-S3. Qualitatively, the Z-score, EWMA, rolling-mean and CUSUM statistics need a few arithmetic operations and constant memory per sample (plus a window buffer for the rolling mean), so they are small enough for a microcontroller. The regression models are the expensive part: Random Forests of up to 200 trees and boosted ensembles need several hundred kilobytes and many comparisons per inference, and the 128–64–32 DANN has roughly $10^{4}$ weights per sensor model. A duty cycle of one wake-up every 5 min leaves ample time for inference, but the cost multiplies with the 13 monitored sensors (one model each). A practical alternative is to run the heavier models on a gateway or server and keep only the residual statistics on the device. Measurements of latency, flash and energy per inference are left to future work.

## 8. Conclusions and Future Work

This paper presented an evaluation of calibration and reference-free drift detection for a heterogeneous environmental sensing platform with incomplete access to reference measurements, based on five devices at two locations and a single injected-fault model.

The calibration experiments showed a clear trade-off between source-domain accuracy and transferability. Random Forest and Gradient Boosting achieved the lowest source-domain errors and are therefore suitable when models can be calibrated for individual deployments. DANN retained a larger fraction of its source performance under transfer (highest mean CTR) and gave the lowest PM_10_ target error under the tested inter-location transfer, and Elastic Net also preserved much of its performance in some transfer scenarios. However, the differences were not statistically significant, DANN’s source accuracy for O_3_ was poor, and for PM_2.5_, no model clearly improved on the raw sensor after transfer; DANN is therefore not established as the generally best approach to multi-modal calibration. O_3_ showed the highest transferability among the evaluated pollutants, whereas NO_2_ and particulate matter were more strongly affected by changes in deployment location; the suggested links to land use and traffic are contextual interpretations and not causal findings, since exact device coordinates and local traffic data were unavailable.

The drift-detection experiments showed that cross-sensor redundancy is a central requirement for reference-free monitoring. Sensors that could be accurately reconstructed from related measurements yielded more stable healthy residuals and clearer responses to synthetic drift; the results demonstrate detection of injected gradual inconsistencies, not of real sensor ageing, and the monitor does not identify which sensor is at fault. Detection performance decreased for sensors with weaker relationships to the remainder of the sensing platform. At the same time, strong correlations may cause a fault in one sensor to affect several residual models, thereby increasing sensitivity to inconsistencies and making fault isolation more difficult.

These results show that calibration and sensor-health monitoring cannot be considered independently of deployment conditions and the sensing platform’s structure. Calibration models should be selected according to the expected degree of domain shift, while residual-based monitoring is most reliable for modalities that exhibit sufficiently stable and informative relationships with other sensors.

Several directions remain for future work. First, the calibration analysis should be extended to longer deployments covering additional locations, seasons and pollutant distributions. A larger deployment would allow the observed differences between device-level and location-level transfer to be tested more rigorously and would provide further insight into the temporal stability of calibration models and the frequency with which recalibration is required.

Second, the drift-detection framework should be evaluated using a broader range of fault conditions. The present evaluation focuses on controlled gradual drift; future experiments should include abrupt bias, scaling errors, increased noise, intermittent faults, stuck-at behaviour and simultaneous sensor failures. Testing multiple fault severities, drift rates and onset times would provide a more complete assessment of detection sensitivity, false-alarm behaviour and detection delay.

Finally, further work is required to move from fault detection towards reliable fault isolation. Residual-signature analysis, structured fault matrices or latent-variable monitoring could help distinguish the faulty sensor from other correlated measurements affected by the same inconsistency. Adaptive thresholds or probabilistic prediction intervals may also improve monitoring for weakly predictable sensors with non-stationary residuals.

Overall, the results demonstrate that heterogeneous sensing platforms can exploit both reference measurements and internal cross-sensor relationships to improve long-term data reliability. Where reference infrastructure is available, deployment-aware calibration can improve measurement accuracy while accounting for domain shift. In cases where references are unavailable, cross-sensor residual monitoring provides a practical mechanism for identifying changes in sensor behaviour. Within the limitations identified above, the proposed framework provides a basis for scalable reliability management in multi-modal environmental sensing deployments.

## Figures and Tables

**Table 1:** Comparison of prior work in sensor calibration, calibration transfer and fault detection.

Study/Domain	Methods	Devices	Duration	Transfer Setting	Target-Domain Labels	Fault Type
Calibration						
Hayward et al. [10]; NO_2_, O_3_, PM_2.5_, PM_10_	MLR, Ridge/Lasso, SVR, RF, GB, MLP, XGB	Multiple LCS types, including AQMesh and Zephyr	Varies by site; up to 20 months	Two-site correction comparison; device/site effects	N/A: no target-domain adaptation	Not evaluated
Gäbel and Hertig [11]; O_3_, PM_2.5_	MLR, Ridge, RF, XGB	2 AELCM units	About 12 months	Pairwise recurrent recalibration at one site	Reference-labelled collocated data	Not evaluated
Mahajan and Helbing [12]; PM_2.5_	Trust-score calibration using accuracy, stability, responsiveness, and consensus	4 real units; 4/9/16-node simulations	48 days real; 300 h simulated	Trust-based consensus; no cross-site transfer	Reference-labelled real data; no target adaptation	Drift/reliability inform calibration; no explicit fault isolation
Nava-Pintor et al. [7]; light/irradiance	Linear and polynomial regression, RF, SVR	1 board; 2 light sensors	About 28 days	Single-site reference regression; no transfer	N/A: no target domain	Not evaluated
Mohanasundaram et al. [13]; PV and meteorological features	Elastic Net feature selection; SVM, ANN, RF, GB	1 PV plant	2020–2023 (3 years)	PV forecasting; not calibration transfer	N/A: prediction task	Not evaluated
**Calibration transfer**						
Dey et al. [14]; PM_2.5_, PM_10_	Semi-supervised adversarial domain adaptation	11 sites	Source: 80 days; target varies	1 source site to 10 target sites	Mostly unlabelled; limited target labels	Not evaluated
Abu-Hani et al. [15]; NO, NO_2_ (O_3_ input)	Global calibration models: MLR, SVR, RF	4 units; 3 sites	About 8 months	Unit transfer within and across sites	Target labels used for evaluation, not fitting	Not evaluated
Cheng et al. [16]; PM_2.5_, temperature, humidity	In-field transfer using distribution similarity and spatial fusion	1000 nodes; 7 reference-equipped	About 10 months	Reference-equipped source nodes to non-reference targets	Unlabelled at target during calibration	Not evaluated
Nowack et al. [17]; NO_2_, PM_10_	MLR, Ridge, RF, GPR	Up to 30 sensor units	About 3 months	Site relocation and transfer evaluated	Target references held out for evaluation	Not evaluated
Malyan et al. [18]; particulate matter	MLR, KNN, RF, GB	22 sites in Delhi	Not reported	Each site-specific model transferred to other sites	Target references for evaluation, not fitting	Not evaluated
**Fault detection and sensor-health monitoring**						
Harkat et al. [19]; O_3_, NO, NO_2_	PCA reconstruction and residual-subspace index	6 stations; 18 channels	1080 samples; time span not reported	N/A: fault detection	N/A: not a calibration-transfer task	Synthetic additive sensor faults
Xu et al. [20]; wind-turbine SCADA	RF normal-behaviour model; adaptive CUSUM	Wind-farm SCADA; sensor count not reported	Not reported	N/A: fault detection	N/A: not a calibration-transfer task	Real operating data; anomaly status unverified
Lin et al. [21]; PM_2.5_	Prediction-based malfunction detection	More than 10,000 sensors	About 8 months	N/A: fault detection	N/A: not a calibration-transfer task	Real malfunctions supported by maintenance records
Kullaa [22]; structural vibration	Bayesian virtual sensing; PCA residuals and extreme-value chart	Simulated bridge sensor network	Not applicable: simulated responses	N/A: damage detection	N/A: not a calibration-transfer task	Simulated structural damage, not sensor faults
Parzinger et al. [23]; HVAC	RF/ARX prediction; statistical residual tests	1 simulated residence	2 months simulated	N/A: fault detection	N/A: not a calibration-transfer task	Synthetic HVAC operating faults
Chahine and Noura [24]; HVAC temperature	LightGBM virtual sensors; Z-scored residuals	LBNL SD-AHU and FCU systems	Not reported	N/A: fault detection	N/A: not a calibration-transfer task	Injected sensor-bias and mechanical faults
Al-Zuriqat et al. [25]; structural temperature	ANN analytical redundancy; detection, isolation, accommodation	10 sensors	About 6 years	N/A: fault detection	N/A: not a calibration-transfer task	Injected sensor faults on real bridge data
Hsu et al. [26]; turbofan	Autoencoder and input-output residuals; healthy-data threshold	C-MAPSS trajectories	Run-to-failure; varies by trajectory	N/A: fault detection	N/A: not a calibration-transfer task	Simulated engine fault/degradation conditions
**This work**; gas, PM, light, UV, T, RH, pressure	MLR, Elastic Net, RF, GB, DANN; cross-sensor prediction with Z-score, CUSUM, EWMA, rolling mean	5 devices; 2 sites	9, 9, 22, 68, 68 days	Within-site and cross-site calibration; no geographic transfer for reference-free channels	DANN uses unlabelled target inputs; references used for evaluation	Injected gradual drift; real sensor ageing not tested

**Table 2:** Device acquisition and processing parameters.

Parameter	Value
Wake interval during battery operation	5 min
Samples per wake	2
Inter-sample interval	5–6 s
Active sampling rate	0.167 Hz
Effective sampling rate	0.0067 Hz
Sampling rate during charging	0.167 Hz
Calibration resolution	1 h
Drift-detection resolution	150 s

**Table 3:** Summary of the datasets used for calibration and drift detection.

Device	Deployment (Days)	Calibration Samples (Valid Hourly Pairs)	Drift SAMPLES
Halle_1	68	1693	261,621
Halle_2	68	1309	437,806
Ukkel_1	9	116	82,792
Ukkel_2	9	143	107,033
Ukkel_3	22	525	350,151

**Table 4:** Hyperparameter search configurations used for the calibration models.

Algorithm	Hyperparameter	Values Evaluated
Linear Regression	None	N/A
Elastic Net	Regularisation strength (en__alpha)	$\left\{1,\;10^{-2},\;10^{-4},\;10^{-6}\right\}$
	L1 ratio (en__l1_ratio)	$\left\{1,\;10^{-2},\;10^{-4}\right\}$
Random Forest	Number of estimators (rf__n_estimators)	{20,60,100,200}
	Maximum tree depth (rf__max_depth)	{3,5,8,None}
Gradient Boosting	Minimum samples per leaf (gb__min_samples_leaf)	{1,4,10}
DANN	Learning rate	$\left\{10^{-3},\;5\times 10^{-4}\right\}$
	Dropout rate	{0.2,0.4}

Mean Absolute Error (MAE) was used as the optimisation criterion. Parameters not listed in the table retained their library default values. For DANN, each learning-rate and dropout combination was trained for 100 epochs. The prediction objective used L1 loss, while the source- and target-domain classifiers used cross-entropy loss.

**Table 5:** Statistical test and ensemble configurations evaluated for residual-based drift detection.

Method	Parameter	Values Evaluated
Z-score	Absolute residual threshold	{2.0,2.5,3.0,3.5,4.0}
CUSUM	Reference value *k*	{0.25,0.50,0.75,1.00}
	Decision interval *h*	{3.0,4.0,5.0,6.0,8.0}
EWMA	Smoothing factor α	{0.10,0.15,0.20,0.25,0.30}
	Control-limit multiplier	{2.0,2.5,3.0,3.5}
Rolling mean	Window length	{10,20,30,50,100} samples
	Absolute mean threshold	{2.0,2.5,3.0,3.5,4.0}
Weighted ensemble	Weight assigned to each test	{0,1,2}
	Decision threshold	{2,3,4,5,6,7,8}
	Minimum persistent alarm run	15 consecutive samples (fixed)

The data were divided into 60% for prediction-model training, 20% for detector tuning, and 20% for held-out evaluation. The held-out evaluation block is one contiguous 20% block placed at 40% of the record, the tuning block is the final 20% of the record, and the remaining data train the prediction model; the split is blocked in time but not purely chronological. Synthetic drift began after 30% of the tuning or evaluation segment.

**Table 6:** DANN architecture and training configuration.

Component	Setting
Feature extractor	Linear(*d*,128)–LayerNorm–ReLU–Dropout–Linear(128,64)–LayerNorm–ReLU–Dropout
Label predictor	Linear(64,32)–LayerNorm–ReLU–Linear(32,1)
Domain classifier (after GRL)	Linear(64,32)–LayerNorm–ReLU–Linear(32,*K*); *K* = number of devices
Loss	L1 (calibration) + cross-entropy (source rows) + cross-entropy (target rows), all with weight 1
GRL coefficient	$\lambda_{p}=2/\left(1+e^{-10p}\right)-1$, *p* = training progress from 0 to 1
Optimiser	Adam, weight decay $10^{-4}$, cosine learning-rate annealing, gradient-norm clipping at 1.0
Batches	128 source rows and 128 target rows per step
Stopping and seeds	Fixed 100 epochs, no early stopping; one run per configuration, seed 42
Hyperparameter grid	Learning rate $\left\{10^{-3},5\times 10^{-4}\right\}$ × dropout {0.2,0.4}

**Figure A1:**
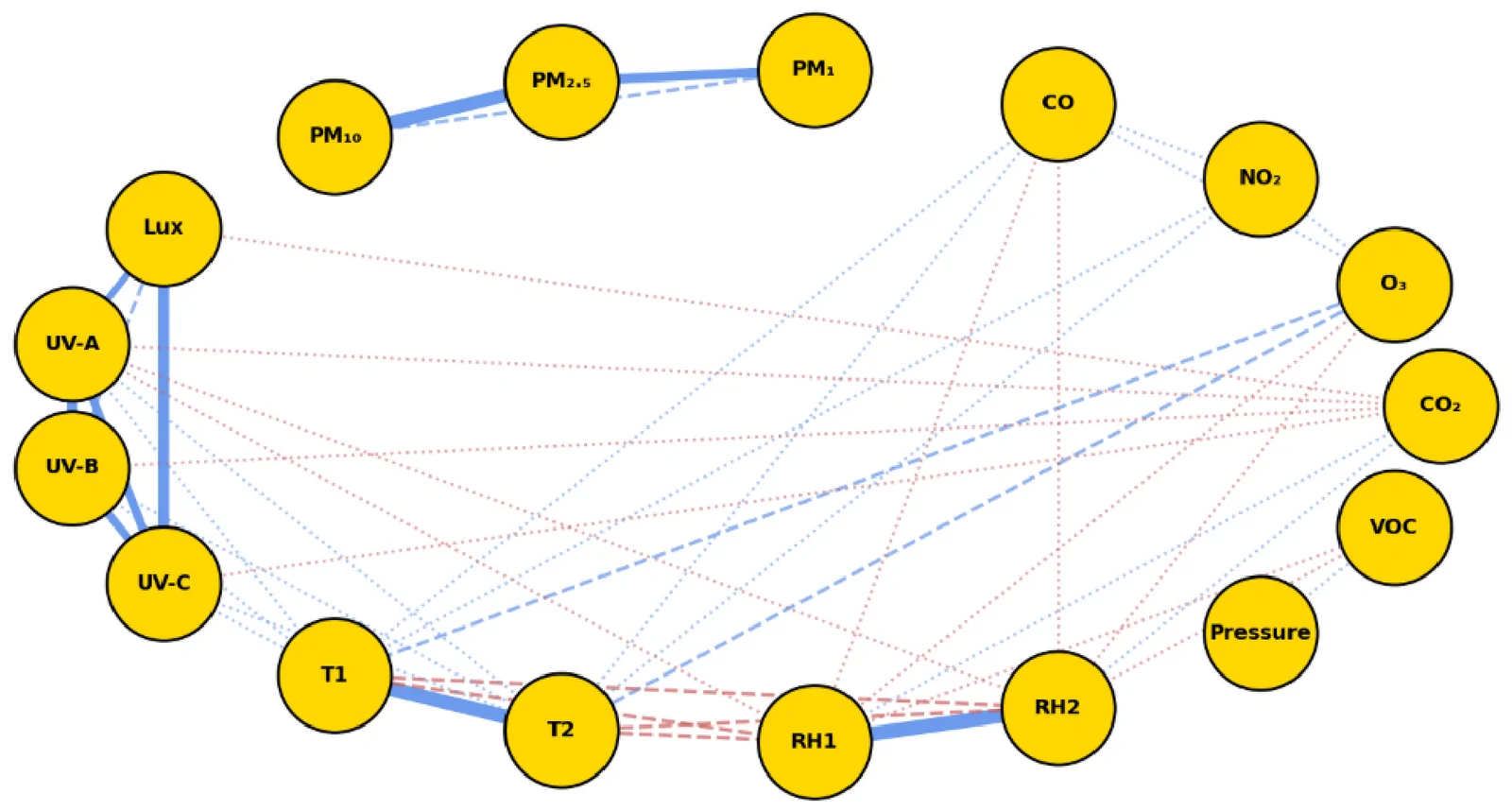
Cross-sensor correlation network aggregated across all available devices. The Pearson Correlation Coefficient is used to assess relationships between sensors. Blue and red edges denote positive and negative relationships; solid, dashed and dotted edges denote strong, medium and weak absolute correlation, respectively. The network is explanatory and should not be interpreted as causal or as a feature-selection proof.

## Data Availability

The source code used for data preprocessing, sensor calibration, calibration-transfer evaluation and reference-free drift detection is publicly available at https://github.com/kishorarvind1999/model_code.git, (Accessed: 24 October 2026). The sensor dataset collected by the five devices is not publicly archived. It is available from the corresponding author on reasonable request, subject to ENACT project restrictions. Regulatory air-quality reference measurements were obtained from IRCEL–CELINE, the Belgian Interregional Environment Agency.

## Appendix A. Complete Calibration Results

This appendix presents the device-specific calibration results underlying the aggregate results in Section 5.1. Source tables report the held-out source MAE. Transfer tables report the target-domain MAE and calibration transfer ratio (CTR) for every ordered source–target device pair. In the transfer tables, each cell is formatted as *target MAE/CTR*. A CTR of one indicates retained source performance, a value below one indicates degradation and a value above one indicates improvement. MAE is in μg/m^3^. For O_3_ and NO_2_, the “Raw” column compares the sensor output in mV directly with the μg/m^3^ reference and is therefore not a meaningful baseline; it is shown for completeness only. Several devices contribute more than one run; the device-balanced aggregate in Appendix C averages repeated runs of the same pair first.

### Appendix A.1. O_3_ Calibration Results

**Table A1:** Device-specific held-out source MAE for O_3_.

Source Device	Raw	MLR	EN	RF	GB	DANN
Halle_1	70.829	30.137	25.720	18.343	15.576	46.580
Halle_2	52.979	107.215	36.098	27.811	28.369	39.104
Ukkel_1	19.314	12.253	10.183	9.731	9.477	15.452
Ukkel_2	19.343	23.699	7.399	9.224	8.475	10.341
Ukkel_3	74.961	22.733	18.633	11.901	10.167	35.272

**Table A2:** Device-specific target-domain MAE and CTR for ordered O_3_ calibration transfers.

Source → Target	MLR	EN	RF	GB	DANN
Halle_1 → Halle_2	43.300/0.697	29.286/0.879	15.455/1.238	15.270/1.069	31.326/1.767
Halle_1 → Ukkel_1	56.495/0.570	46.318/0.638	18.198/1.046	19.805/0.839	–
Halle_1 → Ukkel_2	69.406/0.444	46.000/0.572	17.456/1.074	19.394/0.850	–
Halle_1 → Ukkel_3	47.431/0.640	26.153/1.081	21.706/0.851	23.317/0.658	34.944/1.336
Halle_2 → Halle_1	47.971/2.235	19.946/1.810	17.522/1.587	18.852/1.505	35.019/1.117
Halle_2 → Ukkel_1	213.964/0.501	54.708/0.660	53.136/0.523	59.264/0.479	–
Halle_2 → Ukkel_2	171.858/0.624	48.860/0.739	48.643/0.572	56.859/0.499	–
Halle_2 → Ukkel_3	53.658/1.998	20.199/1.787	22.098/1.259	22.494/1.261	37.499/1.043
Ukkel_1 → Halle_1	46.239/0.265	42.963/0.237	31.442/0.309	29.914/0.317	39.511/0.391
Ukkel_1 → Halle_2	34.556/0.355	35.506/0.287	21.984/0.443	21.361/0.444	29.043/0.532
Ukkel_1 → Ukkel_2	17.272/0.709	11.143/0.914	9.410/1.034	9.968/0.951	13.326/1.160
Ukkel_1 → Ukkel_3	59.980/0.204	48.332/0.211	38.029/0.256	33.997/0.279	38.429/0.402
Ukkel_2 → Halle_1	75.444/0.314	54.551/0.136	28.347/0.325	25.743/0.329	25.399/0.407
Ukkel_2 → Halle_2	40.518/0.585	39.376/0.188	21.993/0.419	21.600/0.392	22.533/0.459
Ukkel_2 → Ukkel_1	28.893/0.820	8.528/0.868	7.998/1.153	6.928/1.223	11.174/0.925
Ukkel_2 → Ukkel_3	55.389/0.428	47.830/0.155	33.512/0.275	31.107/0.272	29.268/0.353
Ukkel_3 → Halle_1	52.668/0.432	39.299/0.474	22.986/0.518	25.045/0.406	23.921/1.475
Ukkel_3 → Halle_2	39.171/0.580	29.188/0.638	22.073/0.539	24.879/0.409	19.754/1.786
Ukkel_3 → Ukkel_1	33.498/0.679	34.410/0.541	27.424/0.434	30.806/0.330	–
Ukkel_3 → Ukkel_2	30.783/0.738	35.707/0.522	24.505/0.486	29.791/0.341	–

### Appendix A.2. NO_2_ Calibration Results

**Table A3:** Device-specific held-out source MAE for NO_2_.

Source Device	Raw	MLR	EN	RF	GB	DANN
Halle_1	5.493	4.584	3.310	3.120	3.099	3.600
Halle_2	8.924	16.872	6.418	4.009	3.831	5.170
Ukkel_1	23.789	4.610	3.939	2.584	2.863	3.224
Ukkel_2	18.280	3.762	3.557	4.670	4.826	4.278
Ukkel_3	5.682	9.041	5.920	4.717	4.132	6.367

**Table A4:** Device-specific target-domain MAE and CTR for ordered NO_2_ calibration transfers.

Source → Target	MLR	EN	RF	GB	DANN
Halle_1 → Halle_2	6.171/0.747	6.221/0.607	4.494/0.707	4.412/0.715	5.084/0.745
Halle_1 → Ukkel_1	10.234/0.454	11.593/0.338	8.088/0.388	9.374/0.324	8.507/0.422
Halle_1 → Ukkel_2	9.236/0.497	10.488/0.374	7.230/0.432	8.084/0.377	7.141/0.503
Halle_1 → Ukkel_3	7.648/0.600	6.189/0.552	5.960/0.516	5.906/0.517	5.547/0.649
Halle_2 → Halle_1	6.094/2.769	3.336/1.924	2.829/1.417	3.027/1.266	3.681/1.405
Halle_2 → Ukkel_1	41.950/0.402	8.787/0.730	8.164/0.491	7.921/0.484	7.309/0.707
Halle_2 → Ukkel_2	35.985/0.469	7.472/0.859	7.246/0.553	6.570/0.583	5.255/0.984
Halle_2 → Ukkel_3	8.561/1.971	5.713/1.123	5.914/0.678	6.026/0.636	5.592/0.925
Ukkel_1 → Halle_1	8.492/0.543	9.950/0.396	8.718/0.296	9.178/0.312	14.381/0.224
Ukkel_1 → Halle_2	10.194/0.452	10.176/0.387	6.496/0.398	6.717/0.426	13.000/0.248
Ukkel_1 → Ukkel_2	7.474/0.617	5.673/0.694	4.774/0.541	4.650/0.616	–
Ukkel_1 → Ukkel_3	15.110/0.305	10.700/0.368	9.417/0.274	9.804/0.292	–
Ukkel_2 → Halle_1	53.996/0.070	7.587/0.469	6.542/0.714	6.440/0.749	9.587/0.446
Ukkel_2 → Halle_2	30.548/0.123	12.754/0.279	5.608/0.833	5.576/0.865	7.515/0.569
Ukkel_2 → Ukkel_1	6.073/0.619	5.314/0.669	4.588/1.018	4.084/1.182	–
Ukkel_2 → Ukkel_3	37.414/0.101	10.797/0.329	7.212/0.648	6.829/0.707	–
Ukkel_3 → Halle_1	20.807/0.435	5.375/1.101	4.466/1.056	4.394/0.940	4.974/1.280
Ukkel_3 → Halle_2	19.665/0.460	5.833/1.015	6.094/0.774	7.472/0.553	6.210/1.025
Ukkel_3 → Ukkel_1	21.347/0.424	7.853/0.754	8.300/0.568	9.055/0.456	–
Ukkel_3 → Ukkel_2	18.604/0.486	6.873/0.861	7.239/0.652	8.657/0.477	–

### Appendix A.3. PM_10_ Calibration Results

**Table A5:** Device-specific held-out source MAE for PM_10_.

Source Device	Raw	MLR	EN	RF	GB	DANN
Halle_1	8.966	6.358	7.161	5.316	5.177	6.492
Halle_2	18.140	26.424	6.804	3.321	3.154	5.896
Ukkel_1	5.695	2.417	2.182	1.220	1.380	3.028
Ukkel_2	4.129	2.946	2.662	2.616	3.419	4.053
Ukkel_3	8.186	34.693	5.395	12.872	6.555	5.144

**Table A6:** Device-specific target-domain MAE and CTR for ordered PM_10_ calibration transfers.

Source → Target	MLR	EN	RF	GB	DANN
Halle_1 → Halle_2	10.729/0.611	8.770/0.958	5.989/0.935	5.946/0.915	6.012/1.080
Halle_1 → Ukkel_1	16.232/0.441	13.915/0.887	6.494/0.847	6.238/0.876	6.481/1.003
Halle_1 → Ukkel_2	20.189/0.343	14.389/0.861	6.356/0.881	6.110/0.903	6.487/1.001
Halle_1 → Ukkel_3	13.544/0.473	9.274/0.772	9.984/0.526	9.778/0.528	9.948/0.654
Halle_2 → Halle_1	18.642/1.417	5.437/1.252	5.258/0.632	4.931/0.640	4.866/1.212
Halle_2 → Ukkel_1	122.944/0.215	5.193/1.310	5.493/0.605	5.893/0.535	4.009/1.471
Halle_2 → Ukkel_2	109.626/0.241	5.100/1.334	5.177/0.642	5.273/0.598	3.238/1.821
Halle_2 → Ukkel_3	34.955/0.756	8.982/0.758	8.907/0.373	9.230/0.342	10.306/0.572
Ukkel_1 → Halle_1	16.653/0.145	6.185/0.353	5.669/0.215	5.701/0.242	6.561/0.461
Ukkel_1 → Halle_2	13.324/0.181	8.721/0.250	6.361/0.192	6.437/0.214	6.182/0.490
Ukkel_1 → Ukkel_2	4.366/0.554	4.027/0.542	3.565/0.342	3.208/0.430	3.573/0.847
Ukkel_1 → Ukkel_3	17.746/0.136	10.428/0.209	9.913/0.123	10.055/0.137	11.126/0.272
Ukkel_2 → Halle_1	30.847/0.095	6.632/0.401	5.710/0.458	6.126/0.558	9.993/0.406
Ukkel_2 → Halle_2	19.357/0.152	8.713/0.306	6.355/0.412	6.594/0.518	8.902/0.455
Ukkel_2 → Ukkel_1	3.031/0.972	3.423/0.778	2.975/0.879	2.756/1.240	3.148/1.287
Ukkel_2 → Ukkel_3	25.813/0.114	12.050/0.221	9.794/0.267	10.425/0.328	12.906/0.314
Ukkel_3 → Halle_1	139.010/0.250	8.216/0.657	31.576/0.408	39.491/0.166	5.252/0.980
Ukkel_3 → Halle_2	128.111/0.271	9.535/0.566	40.915/0.315	52.765/0.124	6.092/0.844
Ukkel_3 → Ukkel_1	137.379/0.253	8.848/0.610	62.834/0.205	70.686/0.093	5.442/0.945
Ukkel_3 → Ukkel_2	124.231/0.279	9.509/0.567	50.822/0.253	58.819/0.111	5.626/0.914

### Appendix A.4. PM_2.5_ Calibration Results

**Table A7:** Device-specific held-out source MAE for PM_2.5_.

Source Device	Raw	MLR	EN	RF	GB	DANN
Halle_1	3.873	3.364	3.931	2.702	2.758	3.525
Halle_2	12.997	7.590	4.922	3.215	3.845	4.075
Ukkel_1	2.449	1.349	1.867	0.702	1.373	2.767
Ukkel_2	2.483	2.554	2.128	2.224	2.882	2.745
Ukkel_3	3.722	9.650	3.527	4.571	2.433	6.126

**Table A8:** Device-specific target-domain MAE and CTR for ordered PM_2.5_ calibration transfers.

Source → Target	MLR	EN	RF	GB	DANN
Halle_1 → Halle_2	6.141/0.550	5.023/0.814	4.037/0.675	3.935/0.706	4.131/0.854
Halle_1 → Ukkel_1	8.403/0.416	6.790/0.716	4.545/0.600	4.579/0.600	4.560/0.774
Halle_1 → Ukkel_2	9.976/0.348	6.897/0.713	4.241/0.639	4.402/0.623	3.733/0.944
Halle_1 → Ukkel_3	7.416/0.462	4.477/0.878	7.978/0.353	9.161/0.322	4.400/0.811
Halle_2 → Halle_1	7.668/0.990	3.336/1.476	3.108/1.035	3.200/1.201	3.117/1.307
Halle_2 → Ukkel_1	40.843/0.186	4.440/1.109	4.379/0.734	4.726/0.814	3.136/1.299
Halle_2 → Ukkel_2	38.098/0.199	3.986/1.235	3.771/0.853	3.898/0.986	2.699/1.510
Halle_2 → Ukkel_3	14.460/0.525	3.664/1.343	3.698/0.870	3.803/1.011	4.220/0.966
Ukkel_1 → Halle_1	11.602/0.116	5.530/0.338	4.625/0.152	5.345/0.257	9.544/0.290
Ukkel_1 → Halle_2	9.647/0.140	7.489/0.249	4.921/0.143	5.076/0.270	8.520/0.325
Ukkel_1 → Ukkel_2	3.675/0.367	3.433/0.544	3.023/0.232	3.042/0.451	3.003/0.921
Ukkel_1 → Ukkel_3	10.753/0.125	7.083/0.264	6.453/0.109	7.324/0.187	8.434/0.328
Ukkel_2 → Halle_1	28.816/0.089	3.485/0.611	3.516/0.633	3.552/0.812	5.560/0.494
Ukkel_2 → Halle_2	17.499/0.146	6.110/0.348	4.634/0.480	4.620/0.624	5.696/0.482
Ukkel_2 → Ukkel_1	2.594/0.985	2.833/0.751	2.643/0.842	2.680/1.075	2.490/1.102
Ukkel_2 → Ukkel_3	21.379/0.119	4.781/0.445	4.555/0.488	4.621/0.624	5.604/0.490
Ukkel_3 → Halle_1	27.645/0.349	4.074/0.866	8.383/0.545	9.070/0.268	3.467/1.767
Ukkel_3 → Halle_2	26.581/0.363	4.879/0.723	11.061/0.413	11.123/0.219	5.297/1.156
Ukkel_3 → Ukkel_1	27.444/0.352	4.502/0.783	13.124/0.348	11.901/0.204	8.520/0.719
Ukkel_3 → Ukkel_2	24.489/0.394	4.410/0.800	11.519/0.397	10.625/0.229	8.988/0.682

## Appendix B. Complete Drift-Detection Results

This appendix presents the device-specific held-out evaluation results for the reference-free drift-detection framework using the RandomForest residual model. Only self-fault experiments, in which gradual drift is injected into the sensor monitored by the corresponding detector, are included. TPR and FPR are reported as percentages. In the device-specific tables, each cell is formatted as *TPR/FPR*. Missing entries indicate that the corresponding channel was unavailable on that device.

**Table A9:** Aggregate held-out drift-detection performance by target sensor.

Target Sensor	TPR Mean ± SEM (%)	FPR Mean ± SEM (%)	*N* Devices
RH_BME68X_	99.3±0.5	2.6±2.4	5
Pressure	2.3±2.3	2.6±2.6	5
Temperature_BME68X_	81.0±17.1	10.7±7.7	5
VOC	64.7±18.2	32.4±14.7	5
CO_2_	69.8±18.4	33.9±16.6	5
CO	99.2±0.6	3.8±3.8	2
RH_SCD40_	81.9±14.7	11.5±6.5	5
Lux	60.3±24.0	2.5±1.5	5
PM_1_	93.0±4.5	4.3±1.8	5
Temperature_SCD40_	90.3±5.4	1.1±0.4	5
UV-A	94.0±5.4	5.2±2.8	3
UV-B	100.0±0.0	4.1±3.6	3
UV-C	100.0±0.0	2.3±2.3	3

**Table A10:** Device-specific held-out TPR/FPR for optical and PM_1_ drift detection.

Target Device	Lux	UV-A	UV-B	UV-C	PM_1_
Halle_1	100.0/0.2	100.0/0.1	100.0/0.0	100.0/0.0	75.3/6.7
Halle_2	98.3/2.3	98.9/9.6	100.0/11.2	100.0/6.8	100.0/0.2
Ukkel_1	3.3/8.2	–	–	–	95.0/0.6
Ukkel_2	0.0/1.8	–	–	–	96.0/9.4
Ukkel_3	100.0/0.1	83.2/6.0	99.9/1.0	100.0/0.0	98.8/4.5

**Table A11:** Device-specific held-out TPR/FPR for temperature and relative-humidity drift detection.

Target Device	Temp._SCD40_	Temp._BME68X_	RH_SCD40_	RH_BME68X_
Halle_1	99.3/0.1	99.4/1.9	91.6/19.8	99.0/0.0
Halle_2	100.0/0.0	100.0/4.6	100.0/4.3	100.0/0.6
Ukkel_1	90.8/2.0	92.7/5.5	97.4/0.1	99.9/12.2
Ukkel_2	69.9/2.0	12.9/41.4	23.3/33.2	100.0/0.2
Ukkel_3	91.4/1.5	100.0/0.0	97.0/0.0	97.6/0.0

**Table A12:** Device-specific held-out TPR/FPR for CO, CO_2_, pressure and VOC drift detection.

Target Device	CO	CO_2_	Pressure	VOC
Halle_1	99.8/0.0	10.3/0.0	0.0/0.0	0.0/0.0
Halle_2	98.6/7.7	100.0/87.1	0.0/0.2	50.6/23.8
Ukkel_1	–	96.6/29.1	11.4/12.8	91.7/38.3
Ukkel_2	–	42.6/52.9	0.0/0.0	99.6/85.6
Ukkel_3	–	99.7/0.2	0.0/0.0	81.7/14.2

## Appendix C. Device-Balanced Calibration Aggregates

**Table A13:** Device-balanced aggregate calibration metrics. Values are the mean over source (source scenario) or source–target (transfer scenarios) device pairs, after first averaging repeated runs of the same pair, with a 95% percentile-bootstrap interval over device pairs in brackets. MAE and RMSE are in μg/m^3^ (O_3_ and NO_2_ “Raw” omitted because the raw output is in mV). Number of pairs: source 5, intra-location 6–8, inter-location 8–12 (pollutant dependent).

Scenario/Pollutant	Metric	MLR	EN	RF	GB	DANN
Source, O_3_	MAE	39.2 [18.1, 74.9]	19.6 [10.8, 28.5]	15.4 [10.0, 22.2]	14.4 [9.2, 21.8]	29.3 [17.4, 40.6]
	RMSE	53.6 [22.2, 108.5]	25.9 [13.8, 38.0]	20.9 [12.6, 32.2]	20.2 [11.4, 33.4]	36.0 [21.3, 50.6]
	$R^{2}$	−5.28 [−12.42, −0.59]	−0.28 [−0.38, −0.16]	0.01 [−0.33, 0.35]	0.10 [−0.29, 0.48]	−1.63 [−2.11, −1.19]
Source, NO_2_	MAE	7.8 [4.3, 12.7]	4.6 [3.6, 5.7]	3.8 [3.1, 4.6]	3.8 [3.2, 4.3]	4.5 [3.6, 5.6]
	RMSE	10.7 [5.9, 18.6]	6.4 [4.9, 8.5]	5.4 [3.9, 7.0]	5.6 [4.3, 7.0]	6.2 [4.9, 7.6]
	$R^{2}$	−1.82 [−4.29, −0.05]	0.00 [−0.27, 0.31]	0.24 [0.03, 0.54]	0.19 [−0.03, 0.46]	−0.04 [−0.45, 0.37]
Source, PM_10_	MAE	14.6 [3.4, 27.4]	4.8 [3.0, 6.7]	5.1 [2.2, 9.0]	3.9 [2.5, 5.6]	4.9 [3.8, 6.0]
	RMSE	19.2 [4.3, 34.0]	6.3 [3.6, 8.9]	9.1 [3.1, 19.0]	5.7 [3.2, 7.9]	6.3 [4.8, 7.8]
	$R^{2}$	−13.60 [−31.98, 0.05]	−0.04 [−0.34, 0.26]	−3.44 [−11.15, 0.59]	−0.05 [−0.71, 0.54]	−0.26 [−0.80, 0.05]
Source, PM_2.5_	MAE	4.9 [2.2, 7.8]	3.3 [2.3, 4.2]	2.7 [1.5, 3.8]	2.7 [2.0, 3.4]	3.8 [2.9, 5.0]
	RMSE	6.5 [3.0, 10.0]	4.2 [2.7, 5.9]	4.2 [2.5, 6.0]	4.0 [2.7, 5.6]	5.1 [4.0, 6.4]
	$R^{2}$	−5.61 [−15.88, 0.27]	−0.45 [−1.42, 0.25]	−1.61 [−5.39, 0.59]	−0.44 [−1.41, 0.36]	−2.30 [−6.43, 0.03]
Intra-location, O_3_	MAE	39.6 [30.3, 48.6]	29.4 [19.8, 39.4]	21.7 [14.9, 28.8]	22.1 [15.0, 28.7]	26.4 [17.9, 34.3]
	RMSE	49.7 [36.7, 62.3]	36.3 [25.1, 47.4]	27.1 [19.5, 34.9]	27.6 [19.6, 35.1]	32.4 [22.0, 42.2]
	$R^{2}$	−3.76 [−5.29, −2.20]	−2.05 [−3.49, −0.45]	−0.68 [−1.51, 0.17]	−0.79 [−1.78, 0.09]	−0.88 [−1.64, −0.10]
	CTR	0.81 [0.50, 1.27]	0.74 [0.44, 1.10]	0.81 [0.50, 1.16]	0.75 [0.43, 1.08]	0.95 [0.59, 1.35]
Intra-location, NO_2_	MAE	14.8 [8.4, 22.3]	7.1 [5.4, 8.8]	6.1 [4.7, 7.5]	6.3 [4.7, 8.0]	4.4 [3.7, 5.1]
	RMSE	18.1 [11.2, 27.5]	9.3 [7.2, 11.5]	8.1 [6.6, 9.8]	8.6 [6.6, 10.7]	6.3 [4.9, 7.8]
	$R^{2}$	−7.40 [−17.48, −1.48]	−0.72 [−1.69, 0.10]	−0.14 [−0.60, 0.24]	−0.29 [−0.83, 0.20]	−0.09 [−0.12, −0.06]
	CTR	0.76 [0.38, 1.37]	0.78 [0.52, 1.15]	0.73 [0.52, 0.98]	0.71 [0.51, 0.95]	1.07 [0.74, 1.40]
Intra-location, PM_10_	MAE	42.7 [11.8, 78.0]	7.8 [5.7, 9.8]	18.9 [5.6, 34.8]	20.9 [5.2, 38.5]	6.6 [4.5, 9.2]
	RMSE	56.6 [25.2, 94.0]	20.3 [8.1, 36.2]	46.2 [19.0, 79.0]	60.2 [17.3, 112.7]	18.9 [6.5, 32.1]
	$R^{2}$	−118.14 [−278.42, −3.76]	−0.43 [−1.03, 0.12]	−86.65 [−211.30, 0.26]	−191.30 [−409.18, 0.28]	0.08 [−0.07, 0.25]
	CTR	0.54 [0.27, 0.86]	0.64 [0.42, 0.88]	0.45 [0.27, 0.67]	0.49 [0.24, 0.76]	0.86 [0.62, 1.09]
Intra-location, PM_2.5_	MAE	13.0 [7.1, 19.4]	4.4 [3.7, 5.3]	6.1 [3.6, 8.9]	5.9 [3.7, 8.3]	5.5 [3.9, 7.3]
	RMSE	16.5 [10.1, 23.1]	7.0 [5.0, 9.1]	11.2 [6.2, 17.0]	10.8 [6.3, 15.9]	8.0 [5.6, 10.6]
	$R^{2}$	−9.02 [−16.66, −2.61]	−0.03 [−0.21, 0.16]	−4.85 [−12.03, 0.19]	−3.87 [−9.67, 0.16]	−0.59 [−1.38, 0.14]
	CTR	0.49 [0.27, 0.72]	0.73 [0.52, 1.01]	0.52 [0.33, 0.73]	0.58 [0.35, 0.87]	0.80 [0.60, 1.03]
Inter-location, O_3_	MAE	75.1 [48.5, 108.2]	40.3 [34.3, 45.7]	27.5 [21.7, 33.7]	29.1 [22.6, 38.0]	29.1 [24.4, 34.1]
	RMSE	88.0 [62.2, 122.0]	48.6 [41.8, 55.7]	33.5 [28.0, 40.5]	35.2 [28.8, 42.6]	37.1 [32.4, 42.0]
	$R^{2}$	−23.19 [−49.40, −5.33]	−3.58 [−4.96, −2.17]	−1.38 [−2.90, −0.27]	−1.80 [−3.80, −0.34]	−0.99 [−1.56, −0.46]
	CTR	0.61 [0.43, 0.88]	0.62 [0.40, 0.89]	0.66 [0.49, 0.83]	0.57 [0.44, 0.74]	0.93 [0.57, 1.30]
Inter-location, NO_2_	MAE	21.4 [13.5, 30.2]	8.5 [7.1, 9.8]	6.7 [6.1, 7.4]	7.0 [6.2, 7.8]	7.9 [6.4, 9.7]
	RMSE	24.9 [16.5, 34.3]	12.7 [9.5, 16.7]	8.5 [7.7, 9.3]	8.8 [7.8, 9.7]	10.4 [8.7, 12.3]
	$R^{2}$	−22.10 [−47.60, −6.20]	−3.07 [−5.65, −1.02]	−0.63 [−1.46, −0.11]	−0.78 [−1.68, −0.18]	−2.05 [−4.24, −0.42]
	CTR	0.54 [0.34, 0.84]	0.64 [0.48, 0.81]	0.59 [0.48, 0.71]	0.56 [0.47, 0.68]	0.67 [0.49, 0.84]
Inter-location, PM_10_	MAE	55.4 [29.3, 83.3]	8.7 [7.3, 10.4]	11.6 [6.4, 18.9]	13.3 [6.4, 23.0]	7.0 [5.7, 8.2]
	RMSE	73.4 [41.5, 109.0]	19.7 [11.6, 29.6]	30.2 [13.3, 48.8]	40.6 [13.3, 74.3]	16.9 [8.7, 27.9]
	$R^{2}$	−138.56 [−263.83, −30.66]	−2.00 [−4.14, −0.36]	−15.13 [−38.56, −0.10]	−43.43 [−107.31, −0.14]	−0.20 [−0.52, 0.04]
	CTR	0.30 [0.21, 0.40]	0.70 [0.52, 0.91]	0.49 [0.38, 0.61]	0.47 [0.33, 0.61]	0.85 [0.62, 1.09]
Inter-location, PM_2.5_	MAE	20.1 [13.7, 26.8]	5.2 [4.4, 5.9]	5.5 [4.4, 6.9]	5.8 [4.5, 7.2]	5.1 [4.1, 6.2]
	RMSE	25.9 [18.5, 34.0]	9.0 [6.9, 11.9]	9.3 [6.3, 12.9]	9.7 [6.6, 13.4]	7.4 [5.8, 9.0]
	$R^{2}$	−27.26 [−46.57, −9.92]	−1.18 [−2.29, −0.38]	−2.16 [−5.10, −0.03]	−2.49 [−6.12, −0.15]	−0.50 [−1.35, 0.03]
	CTR	0.28 [0.20, 0.36]	0.76 [0.56, 0.95]	0.53 [0.40, 0.67]	0.57 [0.41, 0.73]	0.90 [0.66, 1.15]

**Table A14:** Paired Wilcoxon signed-rank tests of pair-level MAE (DANN versus a comparison model), using device-balanced pair means. With only 5–12 pairs per cell, none of the tests would survive a multiplicity correction, so *p*-values are descriptive.

Scenario	Pollutant	DANN vs.	Median MAE Difference	*p*
Source	O_3_	RF	+11.29	0.062
Source	O_3_	GB	+10.74	0.062
Source	O_3_	EN	+5.27	0.062
Source	NO_2_	RF	+0.64	0.125
Source	NO_2_	GB	+0.50	0.312
Source	NO_2_	EN	+0.29	1.000
Source	PM_10_	RF	+1.44	0.625
Source	PM_10_	GB	+1.32	0.312
Source	PM_10_	EN	−0.25	1.000
Source	PM_2.5_	RF	+0.86	0.062
Source	PM_2.5_	GB	+0.77	0.125
Source	PM_2.5_	EN	+0.62	0.438
Intra-location	O_3_	RF	+3.55	0.219
Intra-location	O_3_	GB	+4.34	0.062
Intra-location	O_3_	EN	+2.11	1.000
Intra-location	PM_10_	RF	+0.02	0.945
Intra-location	PM_10_	GB	+0.22	0.844
Intra-location	PM_10_	EN	−0.51	0.250
Intra-location	PM_2.5_	RF	−0.01	0.742
Intra-location	PM_2.5_	GB	−0.06	0.742
Intra-location	PM_2.5_	EN	+0.30	0.383
Inter-location	O_3_	RF	+4.00	0.148
Inter-location	O_3_	GB	+4.31	0.195
Inter-location	O_3_	EN	−7.95	0.312
Inter-location	NO_2_	RF	+0.27	0.266
Inter-location	NO_2_	GB	−0.40	0.677
Inter-location	NO_2_	EN	−0.52	0.380
Inter-location	PM_10_	RF	−0.03	0.677
Inter-location	PM_10_	GB	+0.21	0.910
Inter-location	PM_10_	EN	−1.52	0.151
Inter-location	PM_2.5_	RF	−0.25	0.791
Inter-location	PM_2.5_	GB	−0.34	0.519
Inter-location	PM_2.5_	EN	−0.25	0.733
